# Eukaryotic and archaeal TBP and TFB/TF(II)B follow different promoter DNA bending pathways

**DOI:** 10.1093/nar/gku273

**Published:** 2014-04-15

**Authors:** Andreas Gietl, Phil Holzmeister, Fabian Blombach, Sarah Schulz, Lena Voith von Voithenberg, Don C. Lamb, Finn Werner, Philip Tinnefeld, Dina Grohmann

**Affiliations:** 1 Physikalische und Theoretische Chemie – NanoBioSciences, Technische Universität Braunschweig, Hans-Sommer-Strasse 10, 38106 Braunschweig, Germany; 2 RNAP Laboratory, University College London, Institute of Structural and Molecular Biology, Division of Biosciences, Gower St., London WC1E 6BT, UK; 3 Department of Chemistry, Center for Nanoscience (CeNS) and Center for Integrated Protein Science Munich (CiPSM), Ludwig Maximilian University, Butenandtstraße 11, 81377 Munich, Germany

## Abstract

During transcription initiation, the promoter DNA is recognized and bent by the basal transcription factor TATA-binding protein (TBP). Subsequent association of transcription factor B (TFB) with the TBP–DNA complex is followed by the recruitment of the ribonucleic acid polymerase resulting in the formation of the pre-initiation complex. TBP and TFB/TF(II)B are highly conserved in structure and function among the eukaryotic-archaeal domain but intriguingly have to operate under vastly different conditions. Employing single-pair fluorescence resonance energy transfer, we monitored DNA bending by eukaryotic and archaeal TBPs in the absence and presence of TFB in real-time. We observed that the lifetime of the TBP–DNA interaction differs significantly between the archaeal and eukaryotic system. We show that the eukaryotic DNA-TBP interaction is characterized by a linear, stepwise bending mechanism with an intermediate state distinguished by a distinct bending angle. TF(II)B specifically stabilizes the fully bent TBP–promoter DNA complex and we identify this step as a regulatory checkpoint. In contrast, the archaeal TBP–DNA interaction is extremely dynamic and TBP from the archaeal organism *Sulfolobus acidocaldarius* strictly requires TFB for DNA bending. Thus, we demonstrate that transcription initiation follows diverse pathways on the way to the formation of the pre-initiation complex.

## INTRODUCTION

**Figure 1.**
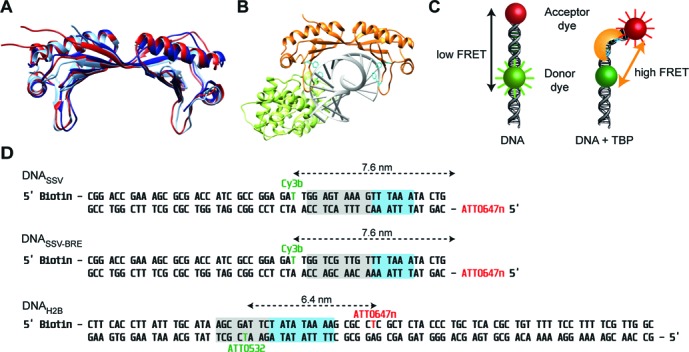
TBP-induced bending of the promoter DNA and DNAs used for the FRET-based single-molecule assay. **(A)** Structural alignment of TBP from *Methanocaldococcus jannaschii* (dark blue), *Sulfolobus acidocaldarius* (light blue) and *Saccharomyces cerevisiae* (red). **(B)** Ternary complex of ScTBP (orange), ScTF(II)B (light green) and DNA. Two sets of two conserved phenylalanines (cyan) are inserted in the minor groove of the DNA backbone (PDB:1C9B). **(C)** Principle of the FRET based bending assay. The TATA-box is framed by a donor (Cy3b or ATTO532) and an acceptor dye (ATTO647n) resulting in a low FRET signal. Once a TBP protein bends the DNA, the distance between the fluorophores is reduced and consequently the FRET efficiency increases. The change in FRET can be analysed on the single-molecule level. **(D)** Promoter sequences used in this study. The upper non-template strand is biotinylated at the 5′ end and internally labelled with Cy3b or ATTO532. The bottom template strand is labelled with the acceptor dye ATTO647n internally or at the 5′ end. The cyan box denotes the TATA-box and the grey box the BRE element. The bending studies for the archaeal model systems were carried out either with the Sulfolobus spindle-shaped virus 1 (SSV) T6 gene promoter (DNA_SSV_) or a mutated version of this (DNA_SSV-BRE_). This DNA contains a mutated BRE element that prevents binding of TFB. Experiments using ScTBP and ScTF(II)B involved the eukaryotic H2B-J core promoter (DNA_H2B_).

Transcription initiation involves the action of two general transcription initiation factors, TATA-binding protein (TBP) and transcription factor B (TFB/TF(II)B), that are crucially involved in the positioning of the ribonucleic acid polymerase (RNAP) at the transcription start site guided by *cis*-regulatory elements. TBP recognizes a sequence motif rich in thymidine and adenine found in the core promoter deoxyribonucleic acid (DNA) termed the TATA-box (DNA_TATA_) (1,2). TFB binds the DNA_TATA_–TBP complex and contacts the promoter DNA at the B-recognition element (BRE) upstream and downstream of the TATA-box resulting in the stabilisation of the complex. Only if both initiation factors are present, the RNAP will be site-specifically recruited to and oriented at the promoter forming the pre-initiation complex (PIC) (3–5). Interestingly, the transcription initiation mechanism is highly conserved among the archaeal-eukaryotic lineage (6–10) reflected not only in the function of the transcription factors (TFs) but also in the high degree of sequence identity among species and an almost identical structural organisation (11,12). The structural conservation is especially pronounced for TBP (Figure 1A and Supplementary Figure S1), a protein with a two-fold, saddle-shaped symmetry that most likely is the result of a gene duplication event (11,13). In eukaryotes, TBP is a part of TF(II)D, a multisubunit complex composed of 13–14 proteins, but minimal PIC assembly can be achieved using TBP and TF(II)B in concert with RNAPII (14–16). Structural studies revealed that TBP induces a substantial bend in the DNA. Bending is caused by the insertion of two sets of conserved phenylalanines into the minor groove of the DNA between the first and last two basepairs of the TATA-box resulting in a widening of the DNA helix and a kink towards the major groove (Figure 1B) (8,17,18). Stopped-flow studies using eukaryotic TBP revealed that fast binding and bending of the DNA by TBP occurs simultaneously (19). Kinetic data further suggest a linear three-step binding scenario for the eukaryotic TBP–DNA_TATA_ interaction and predict two intermediate conformers along the pathway to a final complex. The intermediate and final states have been postulated to vary in their stability but do not show a different bending angle (20–22). Recently, tethered particle-motion measurements confirmed the stepwise bending of the DNA by TBP (23). Similar results were observed using single-molecule fluorescence resonance energy transfer (FRET) where a multitude of FRET states were monitored during the binding process (24). However, after binding, a homogeneous bent state with no influence of the DNA sequence on the bending angle of the DNA_TATA_ was observed (25,26).

Given the importance of this step for differential gene expression, we studied how efficient PIC formation is ensured under varying physiological conditions using (structurally) conserved TBP and TFB/TF(II)B proteins from the eukaryotic and archaeal domain. We monitored the dynamics of the TBP–DNA_TATA_ interaction on the single-molecule level using a FRET signal as readout that allowed us to follow the TBP-induced bending of the promoter DNA (27) (Figure 1C). As TBP binding is inseparably connected to DNA bending, this assay provides information about both complex formation and DNA bending (22). In order to ensure biological relevant temperatures for the archaeal systems, we equipped our single-molecule total internal reflection fluorescence (TIRF) setup with a heating module that allowed temperature-controlled measurements up to 60°C. We tested the impact of environmental factors like temperature, salt concentration and pH value on the formation of the TBP–DNA_TATA_ complex and show that the complex formation is strongly adapted to the physiological conditions found in the respective organism. Interestingly, unlike all other TBP–DNA_TATA_ interactions described so far, DNA bending strictly required TFB when TBP from the crenarchaeal organism *Sulfolobus acidocaldarius* was used. While the archaeal systems showed rapid bending and unbending of the DNA, the eukaryotic TBP–DNA_TATA_ complex was extremely stable with a complex lifetime of minutes. However, this complex is characterized by two interconverting TBP–DNA_TATA_ complexes that differ in their bending angle. TF(II)B is able to shift the equilibrium towards the most bent state which is likely the transcription competent state.

## MATERIALS AND METHODS

### Buffers

Experiments were carried out at room temperature if not indicated otherwise. The buffers used are shown in Table 1. For each experiment, the glycerol concentration was kept constant (surface measurements at 3% and solution measurements at 10% glycerol). The pH listed was measured at room temperature.

**Table 1.** Buffer composition for buffers used in this study

N70	25 mM Na_2_HPO_4_/NaH_2_PO_4_ pH 7.0, 1 M NaCl
N65	25 mM Na_2_HPO_4_/NaH_2_PO_4_ pH 6.5, 1 M NaCl
K72	10 mM K_2_HPO_4_/NaH_2_PO_4_ pH 7.2, 60 mM KCl, 5 mM MgCl_2_, 0.5 mg/ml BSA, 2.5 mM DTT (Dithiothreitol)
M62	MES/NaOH pH 6.2, 60 mM KCl, 5 mM MgCl_2_, 0.5 mg/ml BSA, 2.5 mM DTT
T72	TRIS/HCl pH 7.2, 60 mM KCl, 5 mM MgCl_2_, 0.5 mg/ml BSA, 2.5 mM DTT
T76	TRIS/HCl pH 7.6, 60 mM KCl, 5 mM MgCl_2_, 0.5 mg/ml BSA, 5.0 mM DTT
T82	TRIS/HCl pH 8.2, 60 mM KCl, 5 mM MgCl_2_, 0.5 mg/ml BSA, 2.5 mM DTT
PS	2 mM Trolox, 5 mM protocatechuic acid (pH value of the 100 mM stock was adjusted to each buffers' pH with NaOH or KOH), 50 nM protocatechuate-3,4-dioxygenase

### Proteins

Recombinant TBP and TFB from *Methanocaldococcus jannaschi* (MjTBP and MjTFB) and *Saccharomyces cerevisiae* (ScTBP and ScTF(II)B) were expressed and purified as described previously (28–31). The labelled ScTBP variant was prepared as described in Schluesche *et al.* (25). *S. acidocaldarius* TBP (SaTBP) and C-terminally His-tagged TFB (SaTFB) were expressed from pET30a+ vectors (32) in a heterologous expression system in *Escherichia coli* Rosetta2 (DE3) (Novagen) in enriched growth medium at 37°C for 4 h after induction with 1 mM IPTG (Isopropyl β-D-1-thiogalactopyranoside) . For *S. acidocaldarius* TFB purification, cells were resuspended in N-buffer (25 mM Tris/HCl, pH 8.0, 10 mM MgCl_2_, 100 μM ZnSO_4_, 5 mM beta-mercaptoethanol and 10% glycerol) containing 1 M NaCl supplemented with protease inhibitor (Roche), passed thrice through a French pressure cell press at 16 000 psi. Cell debris was removed by centrifugation (30 000 x g, 30 min) and the supernatant was loaded onto a HisTrap FF cartridge (GE Healthcare) equilibrated in N-buffer containing 500 mM NaCl and 20 mM imidazole. TFB was eluted with buffer containing 250 mM imidazole. The eluate contained both intact TFB1 as well as partial degradation products that most likely contain the C-terminal core domain of TFB1. To remove these partial degradation products, TFB1 containing fractions were combined, diluted with N-buffer to a final NaCl concentration of 200 mM and loaded onto a HiTrap Heparin HP cartridge (GE Healthcare) and eluted with a salt gradient from 200 to 1000 mM NaCl. Fractions containing intact TFB1 were then loaded on a HiPrep 16/60 Sephacryl S-100 HR column in N-buffer containing 500 mM NaCl. TFB1 eluted as a single peak. Protein was concentrated by ultrafiltration (MWCO 10 000, Amicon) and aliquots were stored at −80°C after freezing in liquid nitrogen. For the purification of SaTBP, cells were resuspended in P-buffer (25 mM Tris/acetate pH 8.0, 10 mM MgOAc, 100 μM ZnSO_4_, 5 mM beta-mercaptoethanol and 10% glycerol) containing 300 mM KOAc. After disruption by sonication, cell debris was removed by centrifugation (30 000 x g, 30 min). The cell lysate was incubated for 20 min at 75°C and centrifuged again. The heat-stable supernatant was diluted with P-buffer to 120 mM KOAc and loaded onto a HiTrap Heparin HP cartridge (GE Healthcare). Protein was eluted using a gradient to 1 M KOAc and concentrated by ultrafiltration. Aliquots were snap frozen in liquid nitrogen and stored at −80°C.

The MjTBP^F42A/F59A^ and the SaTFB^C163A/R164E^ mutants were cloned using the QuikChange II site-directed mutagenesis kit (Agilent) and expression and purification of the proteins followed the protocols established for the wild-type protein.

### Synthetic oligonucleotides

In this study, synthetic oligonucleotides derived from strong viral promoters that had been labelled with a donor fluorophore (Cy3B or ATTO532) and an acceptor fluorophore (ATTO647n) were used. The sequences and fluorophore modification sites are listed in Figure 1. In order to study the TBP-induced bending of the double-stranded promoter DNA in the archaeal model systems, the promoter sequence of the Sulfolobus spindle-shaped virus 1 (SSV) T6 gene promoter (DNA_SSV_) was used (5,30). Earlier studies showed that transcription was efficiently driven from this promoter using the transcriptional machinery from the *S. acidocaldarius* as well as *Methanocaldococcus jannaschii* (30,32). Fluorescently labelled DNA strands were purchased from IBA (Göttingen). In order to investigate the contribution of the interaction between TFB and the BRE-element to promoter bending, a modified version of the DNA_SSV_ was used that contained a mutated BRE-element but an intact TATA-box abbreviated with DNA_SSV-BRE_. For ScTBP/TF(II)B experiments, an H2B promoter was chosen (DNA_H2B_) that has been successfully used in singe-molecule studies using ScTBP before (5,25,30,33). Complementary promoter DNA strands (2 μM each) were annealed in 1× TAE (40 mM Tris/acetate pH 8.3, 2.5 mM ethylenediaminetetraacetic acid) with 12.5 mM MgCl_2_ heated up to 95°C and cooled down to room temperature over 1.5 h.

### Surface preparation

Studies on immobilized molecules using a widefield setup were carried out on a PEG (Polyethylene glycol) surface attached to a flow chamber for custom built PRISM-based TIRF microscopy. Quartz slides were thoroughly cleaned with peroxymonosulfuric acid. After drying, the slides were silanized (Aminosilane A0700, amchro Hattersheim) and afterwards PEGylized according to Roy *et al.* (34). Cover slides for solution measurements were cleaned with acetone p.A., EtOH p.A. and deionized water. After a small chamber (Press-to-Seal 2.5 mm with a 3-mm hole stamped out, Sigma Aldrich) had been glued to the cover slide, a solution of 5 mg/ml bovine serum albumin (BSA) in 1× phosphate buffered saline (PBS) was incubated for 10 min. Excessive BSA was removed by washing with 1× PBS. To avoid buffer evaporation during the measurement, the chambers were sealed with a sticky silicone sheet (Press-to-Seal 0.5 mm, Sigma Aldrich).

### TIRF immobilisation assay

For TIRF measurements, the surface was incubated with 0.1 mg/ml neutravidin in 1× PBS for 10 min and washed with 1× PBS. Biotinylated DNAs were immobilized on the glass slide via neutravidin. The glass slide was rinsed with 10 pM DNA solution in 1× PBS and non-bound DNA was removed with 1× PBS. This procedure yielded an immobilized DNA density of approximately one molecule per 4 μm^2^. Afterwards, the flow chamber was flushed with the appropriate measuring buffer. *M. jannaschii* bending assays were carried out in N70 + PS-buffer using 5 nM MjTBP. Assays including MjTFB were performed with a final protein concentration of 3.8 μM. Measurements involving SaTBP and SaTFB were accomplished in N65 + PS-buffer. Since SaTFB is mandatory for promoter bending, it was always used in a concentration of 2 μM. SaTBP was used at 6 μM for room temperature as well as for measurements at 50°C. When ScTBP and ScTF(II)B was used, the promoter DNA (10 pM) was pre-incubated with 30 nM ScTBP (and 500 nM ScTF(II)B) for 20 min at room temperature in T76-buffer to ensure efficient complex formation. The pre-formed complexes were immobilized on the glass surface as described before (35).

### Widefield single-molecule detection and analysis

We used a homebuilt PRISM-TIRF setup based on an Olympus IX71 to perform widefield measurements. Fluorophores were excited with a 532 nm (Coherent Sapphire, Clean-up filter 532/2 MaxLine Semrock, AHF Göttingen) diode laser. The fluorescence was collected by a 60x Olympus 1.20 N.A. water-immersion objective and split by wavelength with a dichroic mirror (640 DCXR, AHF) into two detection channels that were further filtered with a bandpass filter Semrock BrightLine 582/75 in the green channel and a 633 nm RazorEdge long-pass filter (Semrock, AHF) in the red detection range. Both detection channels were recorded by one EMCCD camera (Andor IXon X3, preGain 5.1, gain 250, frame rate varying from 20–100 Hz) in a dual-view configuration (TripleSplit, Cairn, UK) and the videos were analysed by custom-made software based on LabVIEW 2012 64bit (National Instruments). The molecule spots were selected by an automated spot-finder and the resulting transients were filtered with the built in cubic filter of LabVIEW 2012. The fluorescence intensities were background corrected by subtracting the surrounding-pixels' intensity. The transients were also corrected for leakage from the donor into the red detection channel and direct excitation of the acceptor by the 532 nm laser excitation (34). The TBP-induced transition states were analysed with the HaMMy software (assuming two states for the archaeal TBPs and three states for the eukaryotic TBP/TF(II)B complex) developed by the Ha lab and freely available at http://bio.physics.illinois.edu/ (Supplementary Figure S9). In order to heat the sample chamber up to 80°C, a peltier element (HK5163R157L12A from MINCO, Minnesota USA) was glued (J-B WELD 'AutoWELD') to the prism to heat up the sample chamber from above. A detailed description and the voltage–temperature–calibration are shown in the Supplementary Figure S7.

### Confocal measurements

The average concentration of fluorescently labelled DNA was adjusted to less than one molecule per confocal volume (low picomolar range) in order to identify fluorescence bursts from single molecules.

We used a custom built confocal microscope with alternating laser excitation and separate detection of the donor and acceptor fluorescence (36,37). Fluorophores were excited with cw at 532 nm (Sapphire LP 532 nm 100 mW; 170 μW for Cy3b, 190 μW for ATTO532) and with 80 MHz pulsed at 640 nm (LDH-D-C-640, Picoquant, 170 μW for ATTO647n). Alternation of both wavelengths with 100 μs period was achieved using an acousto-optical tunable filter (AOTFnc-VIS, AA optoelectronic). The laser beam was coupled into an oil-immersion objective (60×, NA 1.35 or 100×, NA 1.40, UPLSAPO from Olympus) by a dual-band dichroic beam splitter (Dualband z532/633, AHF). The resulting fluorescence was collected by the same objective, focused onto a 50 μm pinhole, and split spectrally at 640 nm by a dichroic beam splitter (640DCXR, AHF). The photons were detected by two avalanche photodiodes (τ-SPAD-100, Picoquant) with appropriate spectral filtering (donor: BrightLine HC582/75, AHF; RazorEdge LP 532, Semrock; acceptor: Bandpass ET 700/75m, AHF; RazorEdge LP 647, Semrock). The detector signal was processed using a PC card for single-photon counting (SPC-830, Becker&Hickl) and evaluated using custom made LabVIEW 2009 (National Instruments) software. The sample was heated via the objective that was surrounded by a copper ring flushed with water from a temperature controlled water bath (VWR). The effective temperature in the chamber was measured using a Voltcraft PL-120 T2 thermometer equipped with a Pt100 probe for solution (B&B sensors).

### Data evaluation for confocal measurements with alternating laser excitation

Fluorescence bursts from single molecules diffusing through the laser focus were identified by a burst search algorithm applied to the sum of donor and acceptor photons. A single molecule is defined as an event, where, for a minimum of *N* consecutive photons, at least *M* photons are detected within a certain time *T* (parameters used for Cy3b/ATTO647n: *T* = 500 μs, *M* = 30, *N* = 120 and for ATTO532/ATTO647n: *T* = 500 μs, *M* = 10, *N* = 50) (38). Molecules were alternately excited and the fluorescence of donor and acceptor was separately detected. This defines three different photon counts: donor emission due to donor excitation ${F}_{\rm D}^{\rm D}$, acceptor emission due to acceptor excitation ${F}_{\rm A}^{\rm A}$ and acceptor emission due to donor excitation ${F}_{\rm A}^{\rm D}$. After background subtraction, the stoichiometry parameter *S* and the proximity ratio *E* were calculated, where *S* describes the ratio between donor and acceptor dyes of the sample and *E* stands for the proximity ratio as a measure of energy transfer efficiency (36).

\begin{equation*} {E} = \frac{{{F}_{\rm A}^{\rm D} }}{{{F}_{\rm A}^{\rm D} + {F}_{\rm D}^{\rm D} }} \end{equation*}

\begin{equation*} {S} = \frac{{{F}_{\rm A}^{\rm D} + {F}_{\rm D}^{\rm D} }}{{{F}_{\rm A}^{\rm D} + {F}_{\rm D}^{\rm D} + {F}_{\rm A}^{\rm A} }} \end{equation*}

The resulting *E*–*S* histogram was further filtered by the recently introduced ALEX-2CDE (and FRET-2CDE) filter (39) (here: ALEX-2CDE ≤ 12 and if necessary 8 ≤ FRET-2CD ≤ 12). This filter evaluates brightness fluctuations within each burst and therefore eliminates not only the majority of donor-only and acceptor-only labelled molecules, but also fluorophores that exhibit blinking and bleaching. The extraction of *E*-values from the *E*–*S* histograms is illustrated in Supplementary Figure S8.

## RESULTS

### TFs required for promoter DNA bending and the resulting number of detected conformational states for different organisms

**Figure 2.**
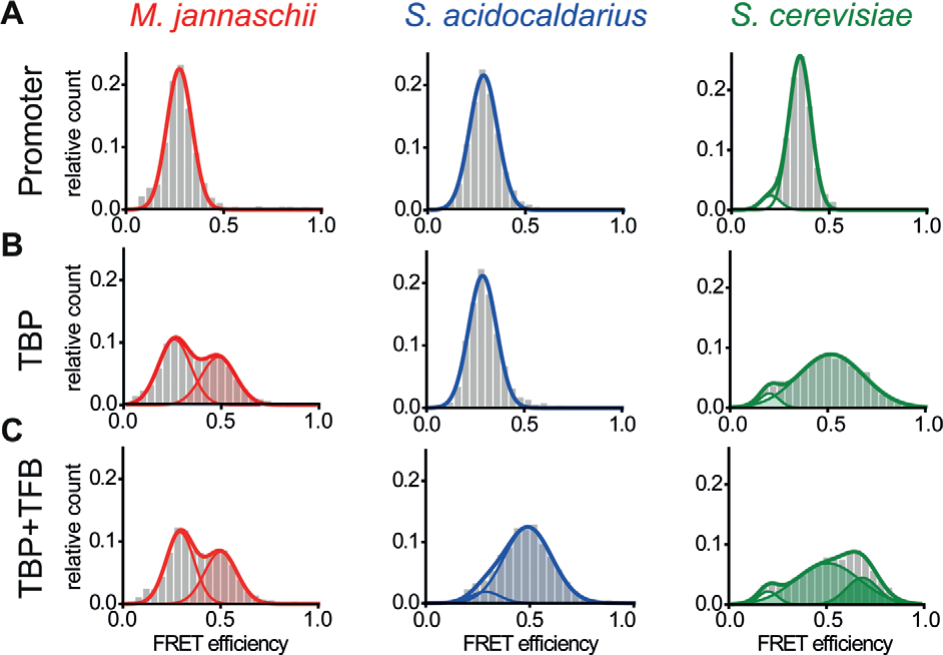
Transcription initiation factors induce promoter DNA bending. **(A)** Donor–acceptor labelled promoter DNA exists in a uniform state with mean FRET efficiencies of 0.25 ± 0.07 and 0.27 ± 0.07 for the DNA_SSV_ in N70 buffer and DNA_SSV_ in N65 buffer, respectively. The eukaryotic promoter DNA_H2B_ shows a major population with a mean efficiency of 0.35 ± 0.06 and a minor subpopulation centered at 0.20 ± 0.05. The minor population remains constant even after addition of TFs and is an inherent property of the labelled DNA construct. The data sets were fitted with a Gaussian fit and the standard deviations are given. **(B)** Addition of MjTBP (1 μM) to the respective DNA resulted in an additional population with a mean FRET efficiency of 0.49 ± 0.09 (shaded area) corresponding to a bent fraction of the promoter while the FRET efficiency of the unbent DNA fraction remains constant at 0.25 ± 0.08. In contrast, addition of SaTBP (6 μM) to the promoter DNA did not cause a shift of the population towards higher FRET efficiencies (0.28 ± 0.07). Incubation of the promoter DNA_H2B_ with ScTBP (20 nM) broadens the FRET distribution towards higher values indicating TBP-induced DNA bending (0.52 ± 0.17). **(C)** While the addition of MjTFB (2 μM) does not alter the FRET distribution significantly (*E*_low FRET_ = 0.27 ± 0.09 and *E*_high FRET_ = 0.48 ± 0.09), the addition of SaTFB (5 μM) facilitates bending of the promoter DNA and an almost complete shift to a high FRET population occurs (*E*_low FRET_ = 0.27 ± 0.09 and *E*_high FRET_ = 0.49 ± 0.11). The presence of ScTF(II)B gives rise to a second high FRET population (*E*_intermediate FRET_ = 0.51 ± 0.16 and *E*_high FRET_ = 0.68 ± 0.08).

TBP and TFB are conserved basal transcription initiation factors found in archaeal and eukaryotic organisms (Supplementary Figure S1). Here, we followed the TBP-induced bending of the promoter DNA using a FRET signal between a donor and acceptor fluorophore attached to the DNA at either side of the TATA-box (Figure 1D). The two DNA arms are getting in close proximity upon bending resulting in an increased energy transfer (Figure 1C). The FRET efficiency of diffusing molecules is determined on the single-molecule level, which allows the identification of different conformational states based on the number of different FRET states detected. Measurements using the free promoter DNA_SSV_ resulted (Figure 2) in homogeneous populations with a mean FRET efficiency of 0.25 and 0.27 using the appropriate buffer conditions for *M. jannaschii* and *S. acidocaldarius*, respectively. The eukaryotic promoter DNA_H2B_ exhibited one main population with a mean FRET efficiency of 0.35 and an inherent minor subpopulation centred at 0.20. Addition of MjTBP to DNA_SSV_ gave rise to a second uniform population with an increased mean FRET efficiency of 0.49 reflecting a conformational change in the DNA upon MjTBP association. Bending of the DNA was strongly dependent on the presence of an intact TATA-box (Supplementary Figure S2). Furthermore, both sets of highly conserved phenylalanine residues that facilitate DNA bending are required for efficient DNA bending (Supplementary Figure S3). Addition of MjTFB did not alter this distribution. Interestingly, no second population was monitored when SaTBP was added to the promoter DNA_SSV_. A second population indicative of a bent DNA state was only observed when both initiation factors, SaTBP and SaTFB, were added simultaneously. In order to find out whether SaTBP binds the DNA without bending, we carried out electrophoretic mobility shift assays to follow complex formation (Supplementary Figure S4). In agreement with the FRET-based assay, we could only detect complex formation when both initiation factors were incubated with the DNA. Supershift analysis using anti-SaTFB and anti-SaTBP antibodies showed that both factors are part of the complex. As TFB makes contacts to both TBP and the BRE element of the promoter DNA, we next investigated which contact is necessary to promote DNA bending. We did not observe a second population corresponding to the bent DNA when DNA_SSV_ was incubated with SaTFB only or when a DNA with a mutated BRE element (DNA_SSV-BRE_) was incubated with SaTFB and SaTBP (Supplementary Figure S5). Similarly, the mutation of an amino acid involved in a crucial ionic interaction that promotes the TBP-TFB contact resulted in the loss of bending activity and concurrently ternary complex formation (Supplementary Figures S4 and S6). This suggests that, in the case of the *S. acidocaldarius* system, TFB is mandatory for promoter DNA bending and requires the contact between TFB and the BRE element as well as the TBP–TFB interaction. However, mutation of the BRE element did not affect the action of MjTBP assuring that the TATA-box is intact even when the BRE element is mutated.

In order to elucidate the molecular similarities and differences between the archaeal and eukaryotic system, we followed complex formation between ScTBP and the promoter DNA. Association of ScTBP with the DNA resulted in a very broad distribution with a mean FRET efficiency of 0.52 (Figure 2B). A wide distribution often indicates fast dynamics between populations with different FRET efficiencies. Hence, this distribution might be indicative of ScTBP–DNA complexes that adopt multiple conformations differing in their bending angle. Addition of ScTF(II)B gave rise to a third population (mean FRET efficiency of 0.68) suggesting that ScTF(II)B either induces a second bent DNA state or stabilizes one of the bent states with high FRET.

### The interaction between promoter DNA and transcription initiation factors is highly adapted to physiological conditions

**Figure 3.**
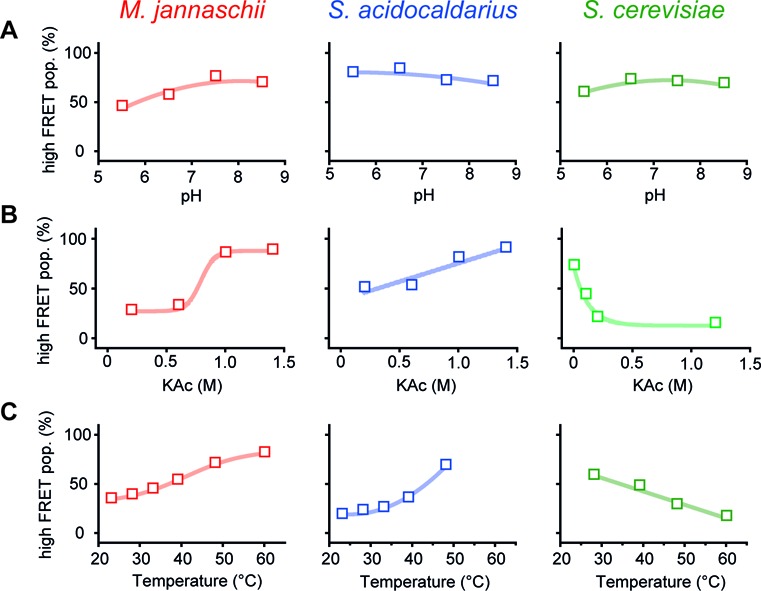
Influence of physiological parameters on TBP-induced bending of promoter DNA. The influence of pH, salt concentration and temperature on the formation of the bent DNA–TBP complex was investigated by comparing the fraction of low to high FRET populations. Measurements involving SaTBP (5 μM) were carried out in the presence of 5 μM SaTFB as this is mandatory for bending. pH and salt dependency studies were performed using confocal solution measurements at 35°C (archaeal proteins) or room temperature (*Saccharomyces cerevisiae*) while the temperature dependence was investigated on immobilized molecules via TIRF microscopy. **(A)** pH dependency: DNA bending is marginally affected by variation of the pH value for *Methanocaldococcus jannaschii* (1 μM MjTBP), *Sulfolobus acidocaldarius* (5 μM SaTBP, 5 μM TFB) and *S. cerevisiae* (30 nM ScTBP). **(B)** Salt dependency: an increase in salt concentration (potassium acetate, KAc) results in a higher fraction of bent DNA for the archaeal systems (1 μM MjTBP, 5 μM SaTBP and 5 μM SaTFB) suggesting a mainly hydrophobic interaction between MjTBP and the DNA. ScTBP-induced DNA bending (30 nM ScTBP in T72) in the eukaryotic system is diminished at higher salt concentrations indicating that the protein–DNA contact is based on electrostatic interactions. **(C)** Temperature-dependency: MjTBP as well as SaTBP/SaTFB (for technical reasons, measurements above 50°C for *S. acidocaldarius* could not be performed) show increased bending efficiency with higher temperature in agreement with their thermophilic growth conditions. The formation of the bent DNA population in the *S. cerevisiae* system is strongly reduced at higher temperatures. The lines in all graphs are only present as a guide to the eye.

The conserved basal transcriptional apparatuses of *M. jannaschii*, *S. acidocaldarius* and *S. cerevisiae* have to function under extremely different physiological conditions, e.g. the optimal growth temperature of the organisms (*T*_Mj_ = 85°C, *T*_Sa_ = 75°C and *T*_Sc_ = 30°C), the intracellular pH (pH_Mj_ = 6.5–7, pH_Sa_ = 6.5, pH_Sc_ = 7.2) (16,40–42) and the cytosolic salt concentration ([K^+^]_Mj_ = 0.9 M, [K^+^]_Sa_ = 24–40 mM, [K^+^]_Sc_ = 160 mM) differ significantly (42–44). Therefore, we next examined how physiological parameters influence the efficiency of promoter recognition and bending. First, we carried out single-molecule fluorescence titrations to determine the dissociation constants for the TBP–DNA_TATA_ interaction for each organism resulting in a *K*_D_ of 48 ± 6, 930 ± 230 and 4.2 ± 1.2 nM for *M. jannaschii*, *S. acidocaldarius* (titration carried out in the presence of 2 μM TFB) and *S. cerevisiae*, respectively (Supplementary Figure S7). These results are in good agreement with a dissociation constant of 22 nM for the MjTBP–DNA_TATA_ interaction determined in ensemble measurements (3,35). Similarly, the binding affinity of ScTBP–DNA_TATA_ has been reported to be in the low nanomolar range, which corresponds well to what we measured (45,46). All further measurements were performed using TBP and TFB concentrations that result in approximately 50% of bent DNA to be able to observe a change in the amount of bent population upon changing the measurement conditions (see figure legends for details). Using single-molecule measurements, we quantitatively assessed the changes in complex formation efficiency as a function of pH, salt concentration and temperature (Figure 3). We found that the interaction between MjTBP, SaTBP/SaTFB and ScTBP and the promoter DNA is not strongly affected by a change in pH as the percentage of the high FRET population is not significantly changed. Next, we investigated the salt dependency (Figure 3 and Supplementary Figure S8) and found that complex formation is enhanced at higher salt concentration for both archaeal model systems. The interaction between ScTBP and promoter DNA is strongly disfavored at high ionic strength. This is true for sodium and potassium cations as well as different counterions (acetate and chloride). We analysed the surface charge distribution of the TBP proteins to rationalize the influence of salt on the archaeal TBP–DNA interaction (Supplementary Figure S9). ScTBP exhibits a positively charged surface area while especially MjTBPs shows a very high density of acidic residues at the surface and a reduced number of charged amino acid at the DNA–protein interface. MjTBP and SaTBP have an excess of 11 negatively charged amino acids over positively charged amino acids whereas, in ScTBP, the positively charged amino acids outweigh by a number of 10. Finally, we tested the influence of temperature on DNA binding and bending by TBP as the chosen archaeal model systems are derived from thermophilic organisms. To this end, we equipped a TIRF setup with a heating device that allowed the measurement chamber to be heated via the prism to temperatures ranging between 20 and 60°C (see ‘Materials and Methods’ section and Supplementary Information) giving us the unique opportunity to record data at elevated temperatures. We found that complex formation strongly depends on temperature and was enhanced by approximately a factor of three when raising the temperature from room temperature to 50°C for the archaeal systems. In contrast, the fraction of the high FRET population decreased by a factor of two when measuring the interaction between ScTBP and the DNA. Notably, the mean FRET efficiencies did not change at high temperatures indicating that archaeal TBPs do not induce a different DNA bending angle at elevated temperatures. Taken together, these data suggest that assembly of the transcriptional machinery at the promoter is optimized to work most efficiently under the physiological conditions present in the respective organism.

### The fraction of bent promoter DNA and the stability of the archaeal TBP–DNA_TATA_ complexes are increased at higher temperatures

**Figure 4.**
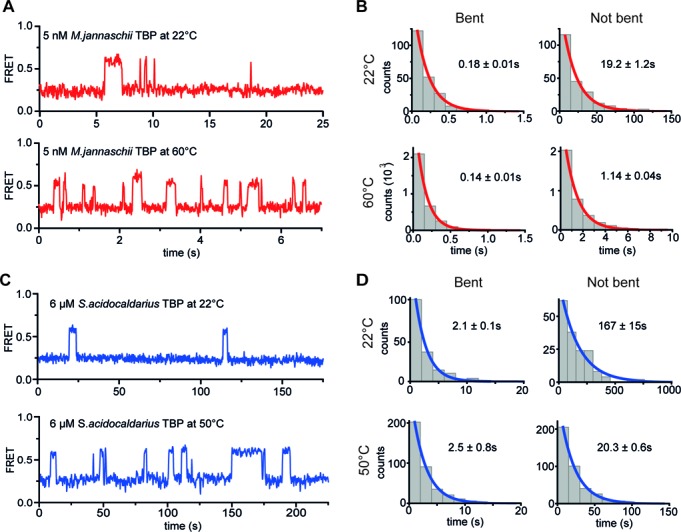
Temperature-dependent dynamics of the archaeal TBP–promoter DNA interaction. Exemplary transients of immobilized promoter DNA molecules upon DNA binding and dissociation and analysis of the kinetics. **(A)** Exemplary traces of DNA_SSV_ in the presence of 5 nM MjTBP at 22 and 60°C. Rapid fluctuations between the low and high FRET states are observed suggesting bending and unbending of the DNA upon rapid association and dissociation of MjTBP. **(B)** Dwell time histograms from a Hidden-Markov-Model analysis of the FRET traces for the respective conditions. The results were fitted with a mono-exponential decay (for details see Supplementary Information). The dwell time and by inference the complex lifetime of MjTBP is almost independent of the temperature (*τ*_22°C_ = 0.18 ± 0.01 s and *τ*_60°C_ = 0.14 ± 0.01 s) while the off-time decreases with increased temperature (*τ*_22°C_ = 19.2 ± 1.2 s and *τ*_60°C_ = 1.14 ± 0.04 s, both with 5 nM MjTBP). **(C)** Exemplary traces of DNA_SSV_ in the presence of 6 μM SaTBP at 22 and 50°C. Similar dynamics can be observed in the presence of SaTBP (6 μM)/SaTFB (2.1 μM). **(D)** The dwell time analysis of transients recorded using SaTBP (in the presence of 2.1 μM SaTFB) yielded comparable results. The dwell time in the bent state remains constant at higher temperature (*τ*_22°C_ = 2.1 ± 0.1 s and *τ*_60°C_ = 2.5 ±0.8 s) while the delay between two bending events is dramatically shortened with higher temperature (*τ*_22°C_ = 167 ± 15 s and *τ*_60°C_ = 20.3 ± 0.6 s). The dynamics immediately stop once the measurement chamber is flushed with fresh buffer indicating that the initiation factors of *Sulfolobus acidocaldarius* and *Methanocaldococcus jannaschii* are not stably bound but that the bending events are caused by different TBP molecules and not by one molecule inducing different bending states.

The temperature-dependent measurements with MjTBP and SaTBP revealed that the equilibrium is shifted towards the bent state of the DNA at elevated temperatures. One possible reason is that the archaeal proteins adopt their 'fully activated' state at high temperatures, which results in a more stable complex formation. We investigated this hypothesis and recorded FRET transients at high and low temperatures (Figure 4A and C). Clear transitions were observed between the bent and unbent conformations. In order to determine the dwell times, we determined and histogrammed the duration of the bent and unbent state of the promoter DNA. Fitting of these data with a monoexponential decay yielded dwell times for the bent and unbent states (Figure 4B and D). Strikingly, the dwell time of the bent state does not increase at elevated temperatures but remains constant for both model systems. From a thermodynamics perspective, the higher thermal energy at elevated temperatures should lead to faster dissociation of the DNA–protein complex. Instead, we observe no reduction of the bent state dwell time for both model systems (*M. jannaschii*: *τ*_22°C_ = 0.18 ± 0.01 s and *τ*_60°C_ = 0.14 ± 0.01 s, *S. acidocaldarius*: *τ*_22°C_ = 2.1 ± 0.1 s and *τ*_50°C_ = 2.5 ± 0.8 s). We explain this result by a larger Gibbs energy of binding for the thermophilic systems at higher temperatures, which stabilizes the complex and compensates for the additional thermal energy. Notably, the lifetime of the unbent state is reduced at higher temperatures by a factor of approximately 17 (*M. jannaschii*: *τ*_22°C_ = 19.2 ± 1.2 s and *τ*_60°C_ = 1.14 ± 0.04 s) and 8 (*S. acidocaldarius*: *τ*_22°C_ = 167 ± 15 s and *τ*_50°C_ =20.3 ± 0.6 s). The increased binding kinetics is at least partly explained by an increased number of collision complexes at higher temperatures so that the overall equilibrium is shifted towards the bent DNA state. However, we cannot rule out the possibility that DNA-associated TBP in an unbent conformation induces the bent state more efficiently at higher temperatures.

### The lifetime of the TBP–DNA_TATA_ complex state differs by several orders of magnitude

**Figure 5.**
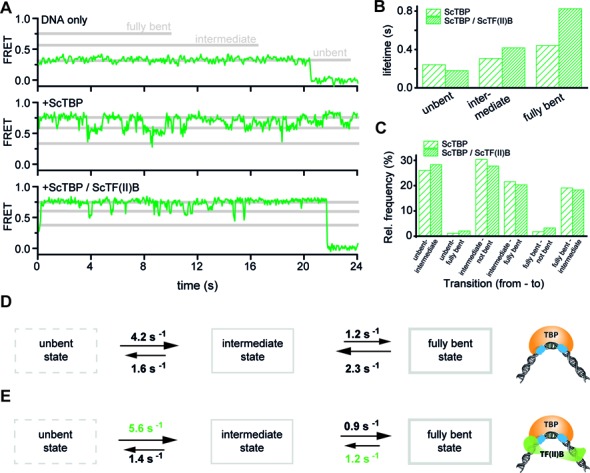
Dynamics of promoter DNA bending in the presence of TBP and TF(II)B from *Saccharomyces cerevisiae*. **(A)** FRET time traces of the promoter DNA only (top), promoter DNA after incubation for 20 min at room temperature with 20 nM ScTBP (middle) and after additional incubation with ScTF(II)B (500 nM). ScTBP remains associated with the promoter DNA for minutes. The traces show different interconverting FRET states exhibiting at least two different bent states, which we assign to the intermediate and fully bent state (*E*_unbent_ = 0.32, *E*_intermediate_ = 0.60 and *E*_fully bent_ = 0.75). **(B)** Dwell time analysis of the different DNA conformations in the presence of ScTBP and ScTBP/ScTF(II)B. Addition of ScTF(II)B leads to a slight increase of the dwell time of the intermediate state (from 0.31 ± 0.02 to 0.42 ± 0.03 s) and more significant increase of the dwell time of the fully bent state (from 0.44 ± 0.04 to 0.83 ± 0.01 s). The lifetime of the unbent state is reduced from 0.24 ± 0.02 to 0.18 ± 0.01 s. See Supplementary Figure S10 for the raw histograms, dwell-time histograms and the mono-exponential fits. **(C)** Relative transition frequency between the three different states shows that the transition from the unbent to the fully bent state and the reverse transition are extremely rare and these transitions are considered as artifacts arising from the limited temporal resolution of 50 ms. This suggests that the three states are connected linearly as shown in the model presented in panels (D) and (E). **(D)** Linear model of the ScTBP action in the absence (D) and presence of ScTF(II)B (**E**). The enhanced lifetime of the fully bent state in the presence of ScTF(II)B is accompanied by a decrease of the transition rate (clearly changed rates highlighted in green) from the fully bent to the intermediate bent state (see Supplementary Figure S10 for details of the transition rate calculation).

**Figure 6.**
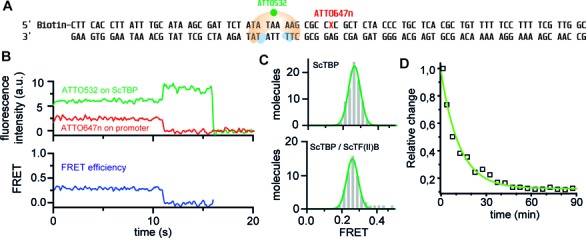
ScTF(II)B does not change the overall architecture of the binary ScTBP–promoter DNA complex. (**A**) In order to measure a FRET signal directly informing about the association of ScTBP with the promoter DNA, a ScTBP mutant was labelled with the donor dye ATTO532 and the acceptor dye ATTO647n was attached to the oligonucleotide through a dT base (position highlighted in red) to the H2B promoter DNA, which can be immobilized via the biotin modification. (**B**) Typical fluorescent transient acquired from the ScTBP–DNA–FRET pair. The fluorescence and hence the FRET efficiency remains constant until the acceptor bleaches (11 s) followed by the bleaching of the donor (16 s). (**C**) FRET efficiency histograms show that the presence of ScTF(II)B (500 nM) does not have any significant influence on the main FRET efficiency (−TF(II)B: *E* = 0.27 ± 0.01, +TF(II)B: *E* = 0.26 ± 0.01 (mean ± SE) ). (**D**) Confocal population analysis reveals a lifetime of the complex of 12.2 ± 1.1 min (see Supplementary methods).

Single-molecule TIRF microscopy measurements allowed us to observe the dynamics of TBP bending and thereby to monitor TBP association and dissociation on immobilized DNA molecules. After flushing of the measurement chamber with TBP, the donor and acceptor fluorescence intensities and the resulting FRET signals were recorded over time and typical fluorescence transients are shown in Figures 4 and 5. A sharp increase in FRET efficiency indicated the binding of TBP that simultaneously results in bending of the DNA (19) and dissociation of TBP resulted in a rapid decrease in the FRET signal. We derived the complex lifetime for the TBP–DNA complex for each model system (see also Supplementary Figure S10) and found that the lifetimes differed by more than one order of magnitude. MjTBP rapidly binds and unbinds with a dwell time in the bent state of 0.18 s. Rapid association and dissociation is also typical for the *S. acidocaldarius* system but the dwell time in the bent state is increased by a factor of 10 (2.1 s). The ScTBP–promoter DNA complexes were extremely long-lived as confocal single-molecule measurements exploiting a FRET signal between a donor-labelled ScTBP variant and the acceptor-labelled DNA showed (Figure 6A). Here, the amount of molecules exhibiting FRET was observed over time and re-association of the TBP molecules was prevented using a large excess of Heparin in the measuring buffer. This allowed us to determine a complex lifetime of 12 min for the ScTBP–promoter DNA complex (Figure 6D).

### The interaction of ScTBP with promoter DNA results in three distinct bent states and follows a linear, stepwise binding mechanism

Interestingly, the ScTBP-DNA interaction does not result in a single high FRET state but rather fluctuates between at least two interconvertible high FRET states (Figure 5) as well as occasional fluctuations to a low FRET state as suspected from the FRET population analysis in Figure 2. In order to avoid rebinding of ScTBP molecules, we flushed the measurement chamber with fresh buffer, which ensured that all dissociated ScTBP molecules were removed. This resulted in comparable FRET traces demonstrating that a single bound ScTBP molecule gave rise to the observed FRET fluctuations. In order to understand the order of transition events we carried out a transition frequency analysis (Figure 5C). The interconversion progressed frequently from the unbent to the intermediate and from the intermediate bent to the high FRET state but also the reverse transition occurred regularly. However, a direct transition from the unbent to the fully bent state and the reverse transition was an extremely rare event, most likely caused by two consecutive transitions during the camera integration time. Therefore, we conclude that the ScTBP-DNA interaction follows a linear, stepwise mechanism (Figure 5D).

### ScTF(II)B shifts the equilibrium towards the fully bent state of the promoter DNA

We next asked whether ScTF(II)B affects one or more of the bent DNA states as suggested from the measurements on diffusing molecules. Therefore, we added ScTBP and ScTF(II)B to the reaction and recorded the FRET signal over time on immobilized molecules (Figure 5A). From the Hidden-Markov-Model analysis, we identify two distinct high FRET states (for further details see Supplementary Information) with mean uncorrected FRET efficiencies (or proximity ratios) of *E*_intermediate_ = 0.60 and *E*_fully bent_ = 0.75, which we termed the intermediate and fully bent states. Dwell time analysis revealed that TF(II)B significantly increased the lifetime of the fully bent state by a factor of 2 (from 0.44 to 0.83 s). The lifetime of the intermediate state was slightly increased (from 0.31 to 0.42 s) whereas the lifetime of the low FRET state was reduced (from 0.24 to 0.18 s) (Figure 5C and Supplementary Figure S10). We derived the transition rates from the dwell times and the time-averaged state distribution (see Supplementary Information) and found that the shift towards the fully bent state was caused by an increased transition rate from the unbent to the intermediate state (*k*_12-TBP_ = 4.2 s^−1^ and *k*_12-TBP/TF(II)B_ =5.6 s^−1^) and a stabilisation of the fully bent state (*k*_32-TBP_ = 2.3 s^−1^and *k*_32-TBP/TF(II)B_ = 1.2 s^−1^) (Figure 5D and E).

Next, we tested whether the stabilising effect of TF(II)B is accompanied by a change in the TBP–promoter DNA complex structure. Here, we employed a donor-labelled ScTBP variant to monitor the intermolecular distance between ScTBP and the acceptor-labelled promoter DNA (Figure 6A). These measurements resulted in a single homogeneous population with a mean FRET efficiency of 0.26. Addition of TF(II)B to the TBP–promoter DNA complex did not alter this distribution. Taken together, these data indicate that TF(II)B stabilizes the ScTBP–DNA complex by shifting the equilibrium towards the fully bent state, which does not necessitate a reorientation of the TBP–promoter DNA complex.

## DISCUSSION

Transcription initiation is a highly regulated step in the transcriptional cycle and involves promoter recognition and subsequent RNAP recruitment by the basal TFs TBP and TFB/TF(II)B. Here, we focused on the formation of the ternary complex composed of the promoter DNA, TBP and TFB analyzing the TBP-induced bending of the promoter DNA on the single-molecule level. Thereby, we could not just add new mechanistic details to the eukaryotic TBP mechanism but could also explore ternary complex formation in the third domain of life shedding light on the evolution of transcriptional regulation at the initiation step.

### The eukaryotic TBP–promoter DNA interaction follows a linear two-step bending mechanism

The interaction between ScTBP and promoter DNA has been studied in detail using ensemble stopped-flow experiments and led to a three-step binding mechanism for human and ScTBP. This model proposes that the final TBP–DNA complex is formed via two intermediate states that differ in their conformation but not in their bending angle (19–22,47). Single-molecule studies using the particle tethered motion (PTM) technique also support the presence of intermediate states but show that one intermediate state differs with respect to the bending angle as compared to the final bent state (23). Single-pair FRET experiments between donor labelled TBP and acceptor labelled DNA by Schluesche *et al.* showed that once ScTBP has firmly bound to the DNA promoter, a single stable complex was observed similar to the data presented in our work (25). In contrast to the PTM analysis, single-molecule FRET data presented by Blair *et al.* identified a single uniformly bent state in addition to the unbent DNA upon human TBP addition (26). Our data on the ScTBP–DNA interaction in the *S. cerevisisae* system provide evidence for a linear, stepwise bending model with a low FRET state, an intermediate and a high FRET state distinguished by a distinct bending angle. We do observe the interconversion between the FRET states even after removal of excessive ScTBP indicating that the transitions between intermediate and high FRET state are caused by a single bound molecule that changes its mode of interaction without dissociation from the DNA.

Mechanistically, the three states could be a result of the initial association of ScTBP with DNA that does not induce a conformational change in the DNA (low FRET population) and the insertion of the first set of phenylalanines into the DNA might cause the intermediate bent state. Finally, intercalation of the second set of phenylalanines might result in the final bent state. This linear three-step-model is supported by the very low occurrence of transitions between the low and the high FRET state and high to low FRET, respectively (Figure 5C). Based on a transition analysis and the action of TF(II)B (see discussion below), we propose that ScTBP first forms an unbent complex with the DNA and progresses via the intermediate to the fully bent status (Figure 5).

Interestingly, the basal transcription factor ScTF(II)B stabilizes the fully bent ScTBP–promoter DNA complex. Our dwell time analysis revealed that ScTF(II)B significantly increases the dwell time in the high FRET state (*τ*_TBP_ = 0.44 s and *τ*_TBP+TF(II)B_ = 0.83 s). Consequently, the transition rate from the high FRET to the intermediate state is decreased. Possibly, this is achieved by preventing the disengagement of the second phenylalanine set of TBP from the DNA. The fully bent state has been proposed to be the transcription competent state (48) and consequently TF(II)B activates transcription via a thermodynamically controlled activation mechanism.

### Molecular mechanisms that lead to ternary complex formation are diversified and adapted to physiological conditions

Even though the structure of the archaeal TBP–TFB–DNA complex is similar in organisation and structure to the eukaryotic counterpart (17,49), our data reveal that the molecular mechanism by which archaeal TBP interacts with the promoter DNA is different to the eukaryotic pathway. Archaeal TBP induces one uniform high FRET population indicating that, in contrast to the eukaryotic system, bending occurs in a single step. In the euryarchaeal model system (*M. jannaschii*), TFB neither influenced the bending angle nor the lifetime of the MjTBP–DNA complex. However, formation of intermediate complexes with the same bending angle but a different conformation in analogy to the eukaryotic pathway cannot be ruled out. The most striking aspect of the archaeal TBP–DNA bending analysis was the finding that, in the crenarchaeal model system of *S. acidocaldarius*, SaTFB is mandatory for SaTBP-induced bending of the promoter DNA which requires a contact of SaTFB with the BRE element in the promoter DNA as well as an interaction with SaTBP. Similarly, the closely related transcription system of *Sulfolobus shibatae* and *Sulfolobus solfataricus* appears also to require TFB for the protection of the TATA-box in DNaseI footprinting assays (16,32,50). Notably, in the transcriptional machinery of eukaryotic RNAPIII system, TBP resides within the TF(III)B complex and bending of the DNA is observed upon addition of TF(III)B on promoters of the type 3 (51–54). A tempting but speculative scenario would be that the co-action of TBP and TFB is the progenitor of the integrated multiprotein transcription factor TF(III)B.

We furthermore found that the interaction between TBP and the promoter DNA exhibits differences in their response to diverse physiological conditions like pH, salt and temperature. In general, the interaction between archaeal TBP and the DNA is driven by hydrophobic interactions between uncharged amino acids and the bases of the DNA whereas the eukaryotic ScTBP–DNA interaction is mediated by electrostatic interactions between charged amino acid sidechains and the phosphate backbone. The salt sensitivity of the eukaryotic TBP–DNA interaction has been shown before (23,55) and high ionic strength disrupts the electrostatic interactions. The stabilising effect of salt for the archaeal system appears to be a general feature at least for thermophilic archaeal systems as it was also described for the archaeal organism *Pyrococcus woesii* (56). Many archaeal proteins are rich in acidic amino acids and have a hydrophobic core which is perceived as an adaptation to high intracellular salt concentrations and high temperatures (57). This high acidic residue content is also found in MjTBP, an organism with high intracellular salt concentration (40), which contains in total an excess of 11 negatively charged amino acids. In comparison, *S. acidocaldarius* also exhibits an overall negative surface charge although the ratio between negatively and positively charged residues is almost balanced (29 versus 28, respectively). High cationic concentrations could lead to the neutralisation of the negative surface charge preventing electrostatic repulsion with the negatively charged DNA. Bergqvist *et al.* also revealed that the interaction between *P. woesii* TBP and DNA is mediated via cation bridges. In the archaeal TBPs, a highly conserved glutamic acid can be found that is not present in mesophilic TBPs. This residue allows the integration of cations in the protein–DNA interface (56) and a mutation of the residue leads to a decrease in affinity at higher salt concentrations. Both high temperature and high salt concentrations stabilize hydrophobic interactions (58,59) and this might explain the hydrophobic nature of the interior of thermophilic TBPs (60). Archaeal organisms counteract high external salt concentration with an increase in internal potassium levels. Thus, hydrophobic interactions allow adaptation to two extreme environmental niches archaeal organisms are able to populate, high temperature and high external salt concentration. In our experiments with TBPs from thermophilic organisms, binding (and bending) times of TBP to the promoter are similar at high and low temperatures. This can be explained by a stabilisation of the complex, which compensates the common Arrhenius-type increase of reaction rate constants with temperature. The energy provided at higher temperatures might be exploited by thermophilic archaea in another way. We found that lower protein concentrations at high temperatures resulted in a higher number of TBP–DNA complexes due, at least in part, to enhanced diffusion. Even low protein levels lead to a comparable TBP-bound fraction at the promoter so that the overall equilibrium is shifted to the bound state at higher temperatures. Hence, reduced protein synthesis is required that saves costly energy gained during a methanogenic lifestyle or by using sulfur as a primary energy source. Interestingly, the complex lifetime of the bent state anticorrelates with the optimal growth temperature of the organisms (*T*_Mj_ = 85°C > *T*_Sa_ = 75°C > *T*_Sc_ =30°C) supporting the idea that enhanced diffusion supports complex formation.

By comparing these three model systems, we found that, even though ternary complex formation involves a shared set of general TFs, each system follows an individual molecular mechanism by which the formation of ternary TBP–TFB–DNA complex is accomplished. Therefore, we conclude that pre-initiation complex formation is a diversified and highly fine-tuned process.

**Figure 7.**
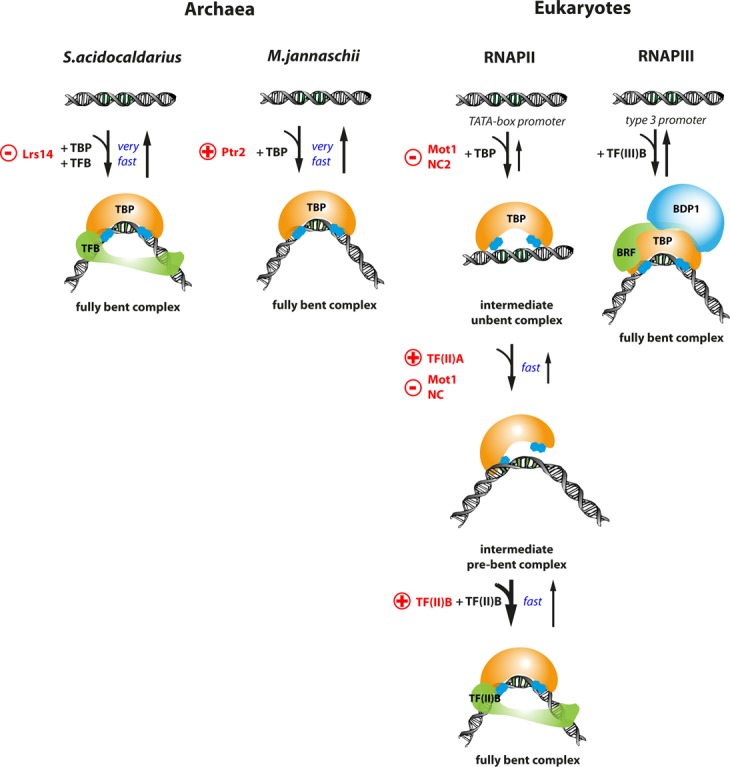
TBP recruitment and DNA bending is a regulatory checkpoint during transcription initiation in archaea and eukaryotic transcription systems. In archaea, the promoter DNA is uniformly bent while eukaryotic TBP induces multiple bent states. However, depending on the archaeal organism, TBP is sufficient (*Methanocaldococcus jannaschii*) or both general TFs, TBP and TFB, are required for promoter DNA bending (*Sulfolobus acidocaldarius*). Transcriptional regulation can be achieved by either preventing or enhancing TBP association with the DNA. Transcriptional activators (indicated with a red plus sign) like Lrs14 and Ptr2 enhance binding of archaeal TBP to the promoter DNA. In contrast, negative regulators like Mot1 and NC2 (indicated by a red minus sign) prevent archaeal TBP interaction with the DNA. The general eukaryotic transcription factor TF(II)A stabilizes the eukaryotic TBP–DNA complex as does ScTF(II)B. ScTF(II)B exerts its stabilising and potentially regulatory effect by a shift in binding equilibrium towards the transcriptional active, fully bent DNA state. ScTF(II)B acts in a sequential manner to eukaryotic TBP in the transcriptional system of RNAPII while DNA bending requires co-action of TBP and TFB (called Brf in the RNAPIII system) in the RNAPIII system and *S. acidocaldarius*. TBP and TFB are inherently co-acting in the transcriptional apparatus of the eukaryotic RNAPIII system as they are part of the multidomain complex TF(III)B.

It has been shown that gene-specific TFs in the archaeal system (Ptr2 and Lrs14 (15,61)) and eukaryotic global TFs (Mot1 and NC2 (25,62)) regulate transcription by directly preventing or enhancing the recruitment of TBP to the promoter DNA. In a similar fashion, TF(II)A has been shown to influence the TBP-DNA interaction by increasing the kinetic stability of the complex and relieves negative transcriptional activities of the TAF (TBP-associated factors) subunits of TF(II)D (63). Acting at the first step in PIC formation, these mechanisms can be perceived as the initial level of transcriptional regulation. Given the long complex lifetime of the eukaryotic ScTBP–DNA complex, it seems plausible that mechanisms for activation or removal of DNA-associated ScTBP are in place that would allow rapid gene regulation. Here, we show that the general transcription factor ScTF(II)B might fulfill this activation function as it shifts the equilibrium towards the fully bent DNA, which has been proposed to be the transcriptionally active configuration (48). Already assembled ScTBP at the promoter can thereby quickly convert to a transcriptionally active PIC when ScTF(II)B associates and stabilizes the fully bent DNA topology. Based on our data, promoter DNA bending, in addition to ScTBP recruitment, is a second regulatory checkpoint during transcription initiation (Figure 7).

Stabilisation of the TBP–DNA complex by TFB does not seem to be a general feature of TFBs in all species as we have shown for the *M. jannaschii* system that MjTFB does not exert any influence on complex lifetime or the bending angle. The short complex lifetime of the archaeal TBP–DNA complex allows regulation of transcription initiation by gene-specific factors directly at the TBP recruitment stage as TBP is not permanently bound to the DNA. Numerous archaeal organisms encode multiple homologues of the TBP and TFB proteins (64) and it has been speculated that alternative combinations of these factors activate transcription of specific genes (65). *S. acidocaldarius* encodes one SaTBP and three SaTFB proteins (66) and here, the co-action of SaTBP and SaTFB is required for promoter DNA bending. Thus, the combination of a SaTBP/SaTFB pairing that is bending competent in dependence of *cis*-regulatory elements might be an additional mechanism that mediates specificity among TATA-containing promoters in archaeal organisms.

Notably, PIC formation and transcriptional activation is not uniform in eukaryotes either and the concerted action of TBP and TFB in analogy to *S. acidocaldarius* is characteristic for the RNAPIII transcription system as well. RNAPIII-directed transcription is aided by two multiprotein factors termed TF(III)B and TF(III)C (67). TF(III)B is composed of TBP, the TF(II)B-factor related factor and B double prime (Figure 7). Here, TF(III)B is necessary and sufficient to direct transcription from the TATA-containing U6 gene promoter while transcription of the TATA-less 5S ribosomal RNA and transfer RNA genes is co-directed by the general transcription factor TF(III)C. Interestingly, TF(III)B sharply bends the DNA even on TATA-less genes supporting the idea that DNA bending is a prerequisite for transcriptional activation (53,54).

## SUPPLEMENTARY DATA

Supplementary Data is available at NAR Online.

SUPPLEMENTARY DATA
