# Supplementary material for: Eukaryotic and archaeal TBP and TFB/TF(II)B follow different promoter DNA bending pathways
Source: Nucleic Acids Res. 2014 Apr 15;42(10):6219–31. doi: 10.1093/nar/gku273 (PMC4041446; doi:10.1093/nar/gku273)
Supplement: SUPPLEMENTARY DATA [file supp_42_10_6219__index.html]

Eukaryotic and archaeal TBP and TFB/TF(II)B follow different promoter DNA bending pathways — Eukaryotic and archaeal TBP and TFB/TF(II)B follow different promoter DNA bending pathways — SUPPLEMENTARY DATA 

# Eukaryotic and archaeal TBP and TFB/TF(II)B follow different promoter DNA bending pathways

## SUPPLEMENTARY DATA

**Files in this Data Supplement:**

- SUPPLEMENTARY DATA
