# Supplementary material for: Eukaryotic and archaeal TBP and TFB/TF(II)B follow different promoter DNA bending pathways
Source: Nucleic Acids Res. 2014 Apr 15;42(10):6219–31. doi: 10.1093/nar/gku273 (PMC4041446; doi:10.1093/nar/gku273)
Supplement: SUPPLEMENTARY DATA [file supp_gku273_nar-03206-f-2013-File009.docx]

**Supplementary Information for**

**Eukaryotic and archaeal TBP and TFB/TF(II)B follow different promoter DNA bending pathways**

Andreas Gietl, Phil Holzmeister, Fabian Blombach, Sarah Schulz, Lena Voith von Voithenberg,^,^ Don C. Lamb, Finn Werner, Philip Tinnefeld and Dina Grohmann

[1 Sequence alignment of TBPs and TFBs 2](#_Toc382918192)

[Supplementary Figure S1 2](#_Toc382918193)

[2 Variation of TATA box sequence for promoter DNA bending studies 3](#_Toc382918194)

[Supplementary Figure S2 3](#_Toc382918195)

[3 Effect of MjTBP phenylalanine mutation on promoter DNA bending 4](#_Toc382918196)

[Supplementary Figure S3 4](#_Toc382918197)

[4 Promoter DNA binding by TBP and TFB from *M.jannaschii* and *S.acidocaldarius* 5](#_Toc382918198)

[Supplementary Figure S4 5](#_Toc382918199)

[5 TFB action depends on the BRE element in the promoter DNA 6](#_Toc382918200)

[Supplementary Figure S5 6](#_Toc382918201)

[6 Disruption of the SaTFB-SaTBP interface inhibits promoter DNA bending 7](#_Toc382918202)

[Supplementary Figure S6 7](#_Toc382918203)

[7 Single-molecule titration of transcription factors and promoter DNA 8](#_Toc382918204)

[Supplementary Figure S7 8](#_Toc382918205)

[8 Influence of NaCl concentration on TBP-induced bending of promoter DNA 9](#_Toc382918206)

[Supplementary Figure S8 9](#_Toc382918207)

[9 Representation of the surface charge of the TBP proteins 10](#_Toc382918208)

[Supplementary Figure S9 10](#_Toc382918209)

[10 *S.cerevisiae* TIRF data and transition state calculation 11](#_Toc382918210)

[Supplementary Figure S10 11](#_Toc382918211)

[11 Supplementary Methods 13](#_Toc382918212)

[11.1 Electrophoretic mobility shift assays (EMSA) 13](#_Toc382918213)

[11.2 Installation and calibration of the heating unit at the TIRF microscope 14](#_Toc382918214)

[Supplementary Figure S11 14](#_Toc382918215)

[11.3 Confocal Analysis 15](#_Toc382918216)

[Supplementary Figure S12 16](#_Toc382918217)

[11.4 TIRF analysis 17](#_Toc382918218)

[Supplementary Figure S13 17](#_Toc382918219)

[12 Supplementary References 18](#_Toc382918220)

# Sequence alignment of TBPs and TFBs

**
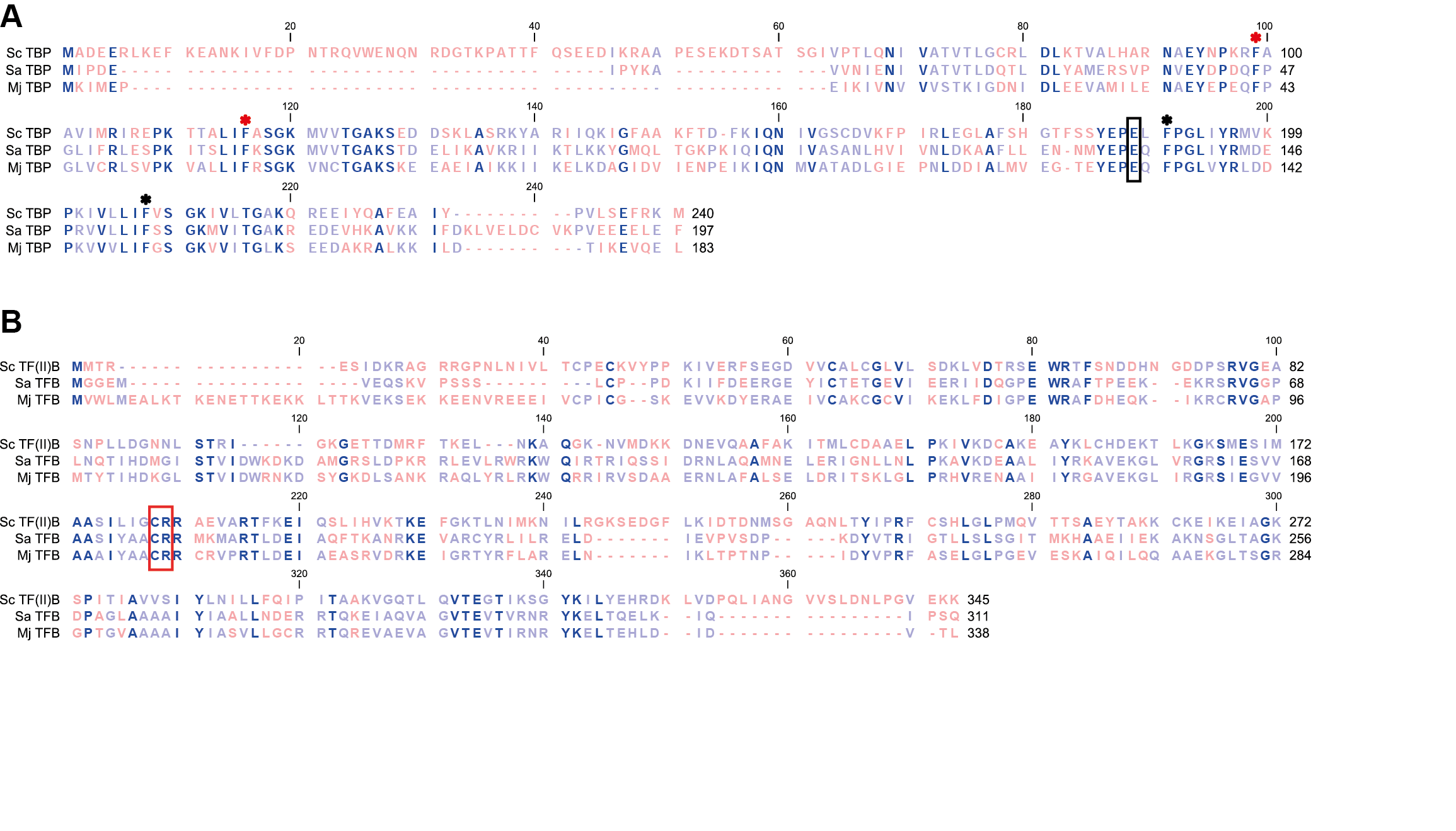
**

Supplementary Figure S1 related to Figure 1.

**Sequence alignment of TBPs and TFBs.** Amino acid sequence alignment of archaeal and eukaryotic **(A)**  TBP and **(B)** TFB/TF(II)B from the chosen model organisms (Sc: *Saccharomyces cerevisiae*, Mj: *Methanocaldococcus jannaschii*, Sa: *Sulfolobus acidocaldarius*. Highly conserved residues are shown in dark blue, non-conserved residues in red. The highly conserved phenylalanine residues involved in DNA bending are indicated with stars. Highly conserved residues that contribute to the TBP-TFB interaction are boxed. For experiments described later, the phenylalanines indicated with the red stars were mutated in MjTBP (**see Figure S5**) and the residues highlighted in the red box were mutated in SaTFB (see **Figure S7**). The alignment has been created using the CLC Sequence Viewer 6.3 software (www.clcbio.com).

# Variation of TATA box sequence for promoter DNA bending studies


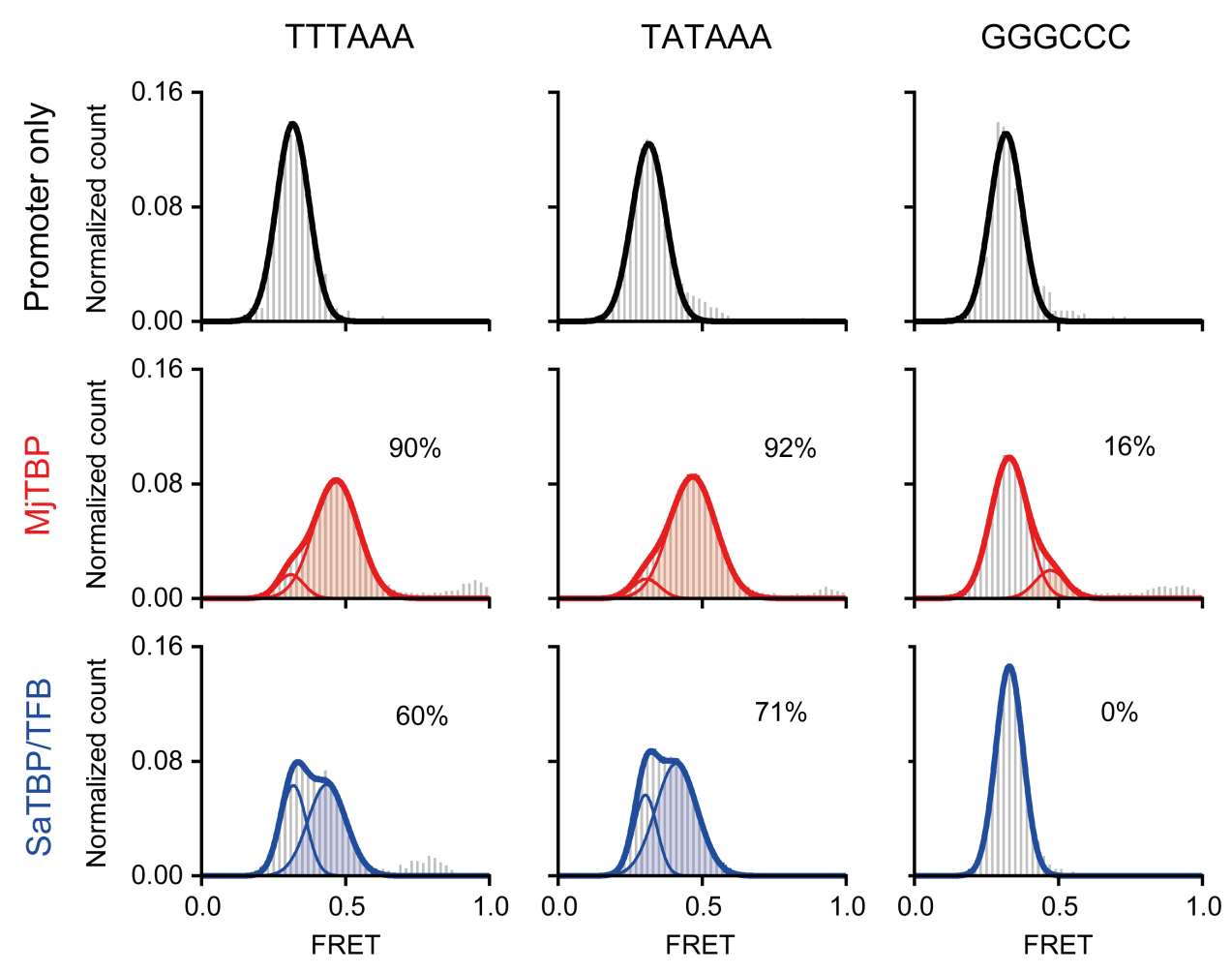


Supplementary Figure S2 related to Figure 2**.**

**Influence of TATA-box sequence on promoter DNA bending efficiency.** TBP-induced bending is dependent on the TATA-box sequence. The presence of the TATA-box element (TTTAAA) of the strong viral promoter SSV T6 in the promoter DNA (used throughout this study) leads to efficient DNA bending. For comparison, the consensus TATA-box motif found in *M.jannaschii* ([1](#_ENREF_1)) is comparably efficient (MjTBP) or slightly more efficient (SaTFB/TBP) in DNA bending as observed for the TTTAAA sequence. In order to demonstrate the sequence-specificity of TBP-induced bending, the TATA-box was replaced by a completely TA-deficient control sequence (GGGCCC). TBP-induced promoter DNA bending was strongly reduced for this sequence in the case of MjTBP and completely lost for SaTBP/SaTFB.

All assays were performed under the same conditions / concentrations as for Figure 2.

# Effect of MjTBP phenylalanine mutation on promoter DNA bending


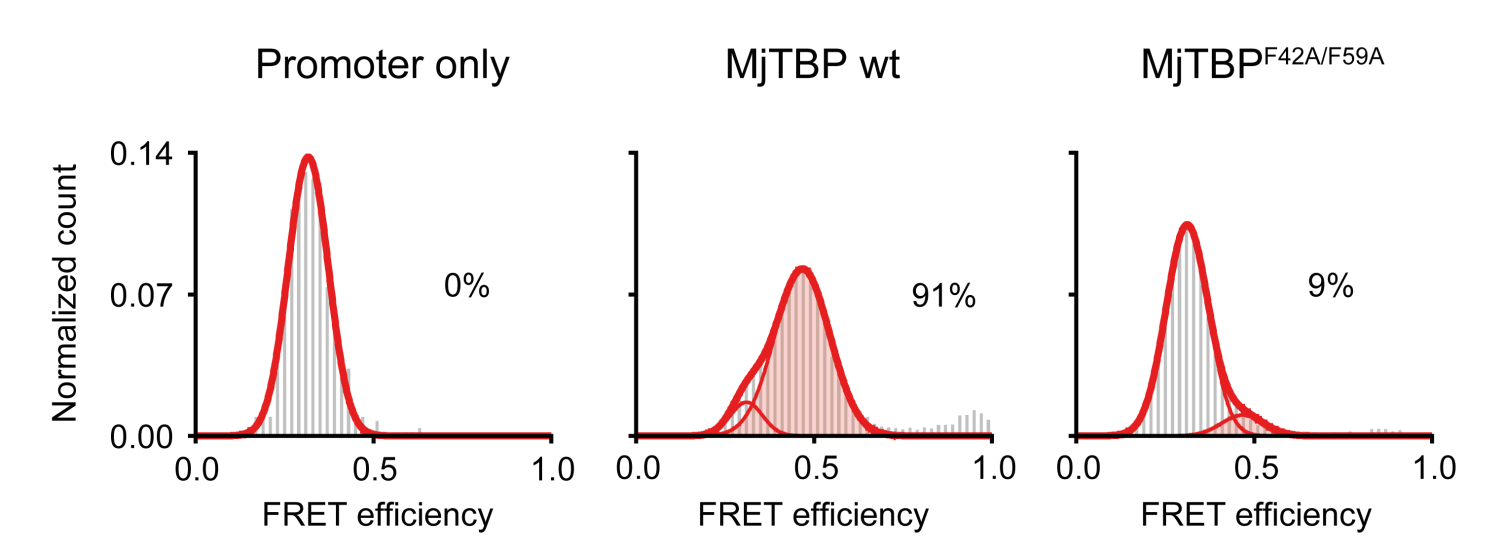


Supplementary Figure S3 related to Figure 2**.**

**Characterization of a bending-impaired TBP mutant.** Bending of the promoter DNA is caused by the insertion of two sets of phenylalanine residues into the DNA. Mutation of one set of these phenylalanines (double mutant MjTBP^F42A/F59A^) results in almost complete loss of bending ability of MjTBP.

All assays were performed under the same conditions / concentrations as for Figure 2.

# Promoter DNA binding by TBP and TFB from *M.jannaschii* and *S.acidocaldarius*

**
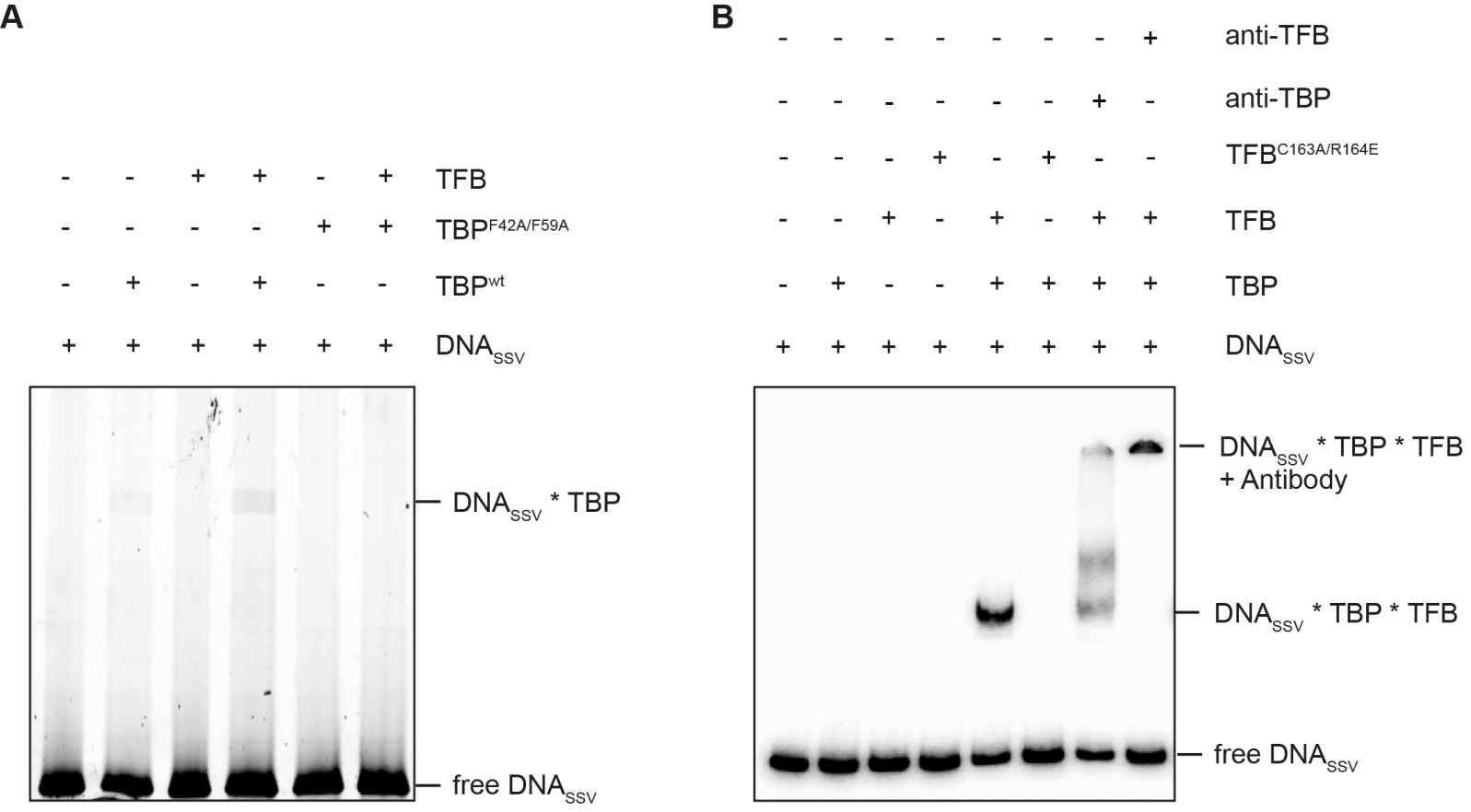
**

Supplementary Figure S4 related to Figure 2**.**

**Promoter DNA binding by TBP and TFB.** EMSA using fluorescently or radiolabelled promoter DNA derived from the Sulfolobus spindle-shaped virus 1 (SSV) T6 gene promoter and TBP and TFB from either **(A)** *M.jannaschii* or **(B)** *S.acidocaldarius*. Addition of MjTBP leads to the formation of a binary complex. No formation of a ternary complex with different migration behaviour was observed upon addition of MjTFB. Complex formation is not visible when one of the phenylalanine sets in MjTBP is mutated (TBP^F42A/F59A^ mutant). In contrast, a detectable complex is only formed when both SaTBP and SaTFB are added to the promoter DNA indicating that SaTFB is mandatory for TBP recognition of the promoter DNA. Mutation of two highly conserved residues (SaTFB^C163A/R164A^) at the interface between SaTBP and SaTFB disrupts the ternary complex formation (see also **Supplementary Figure S6**). Supershift analysis using anti-SaTFB or anti-SaTBP antibodies (**B**) revealed a clear supershift of the ternary complex indicating that both, SaTFB and SaTBP, are part of the observed ternary complex. Preimmune serum as control did not cause any supershifts (data not shown).

# TFB action depends on the BRE element in the promoter DNA


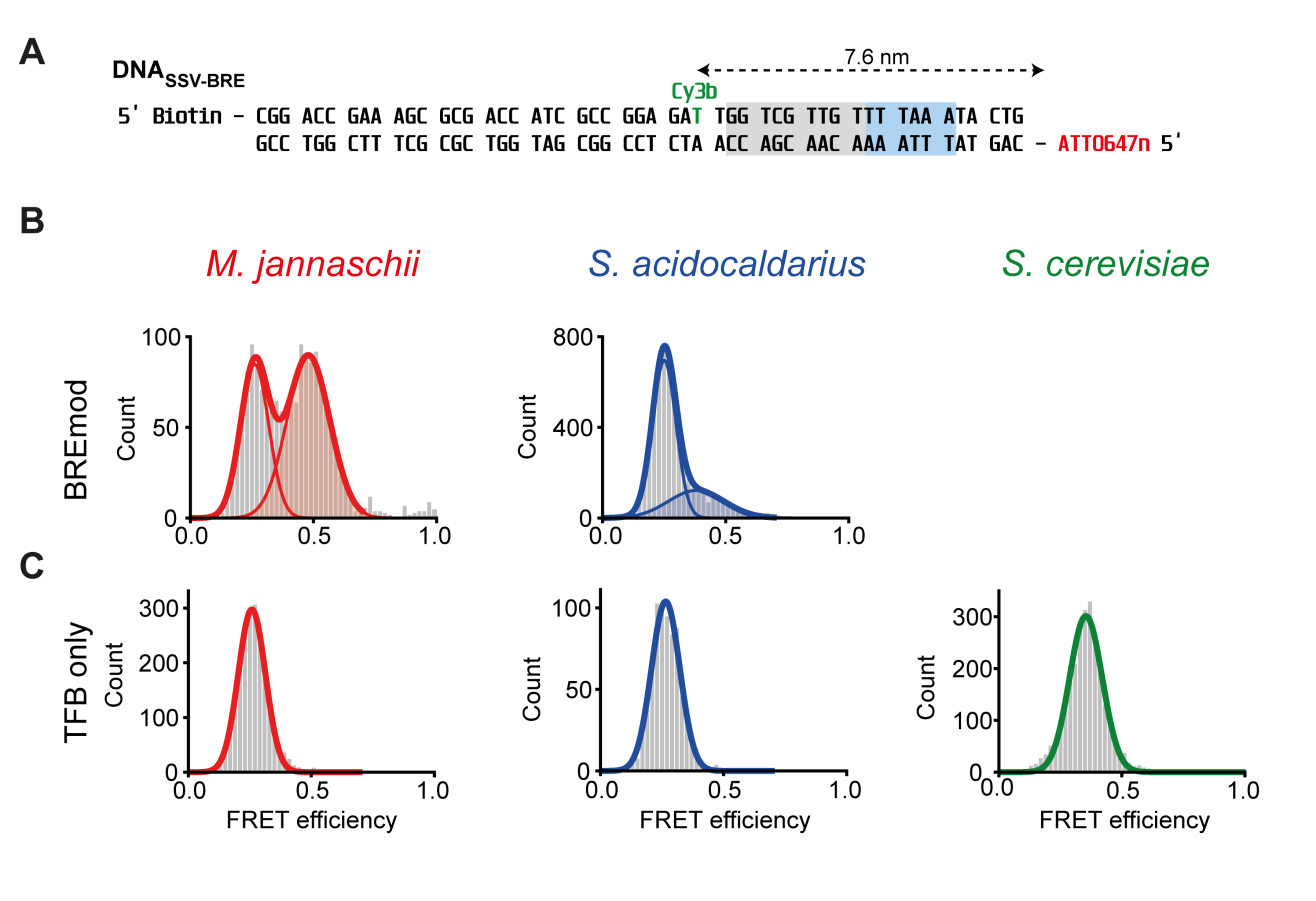


Supplementary Figure S5 related to Figure 2.

**Experiments with mutated BRE element. (A)** Sequence of SSV promoter with modified BRE element (grey) and still intact TATA box (cyan). **(B)** Single-molecule FRET histograms of DNA_SSV-BRE_ in the presence of TBP and TFB for *M. jannaschii* and *S. acidocaldarius.* While the modified BRE element does not significantly influence the MjTBP + MjTFB bending efficiency (high FRET population without BRE modification: 48% and with BRE modification: 62%), the mutation of the BRE element prevents SaTFB to facilitate the bending as there is only a very small high FRET fraction recorded (high FRET population wildtype DNA: 92% and with mutated BRE element: 29%). **(C)** Single-molecule FRET histograms of DNA_SSV-BRE_ in the presence of MjTFB, SaTFB or ScTF(II)B only. None of the TFBs does cause any shift of the FRET efficiency by itself, and hence TFB is not able to bend the promoter.

All assays were performed under the same conditions / concentrations as for Figure 2.

# Disruption of the SaTFB-SaTBP interface inhibits promoter DNA bending


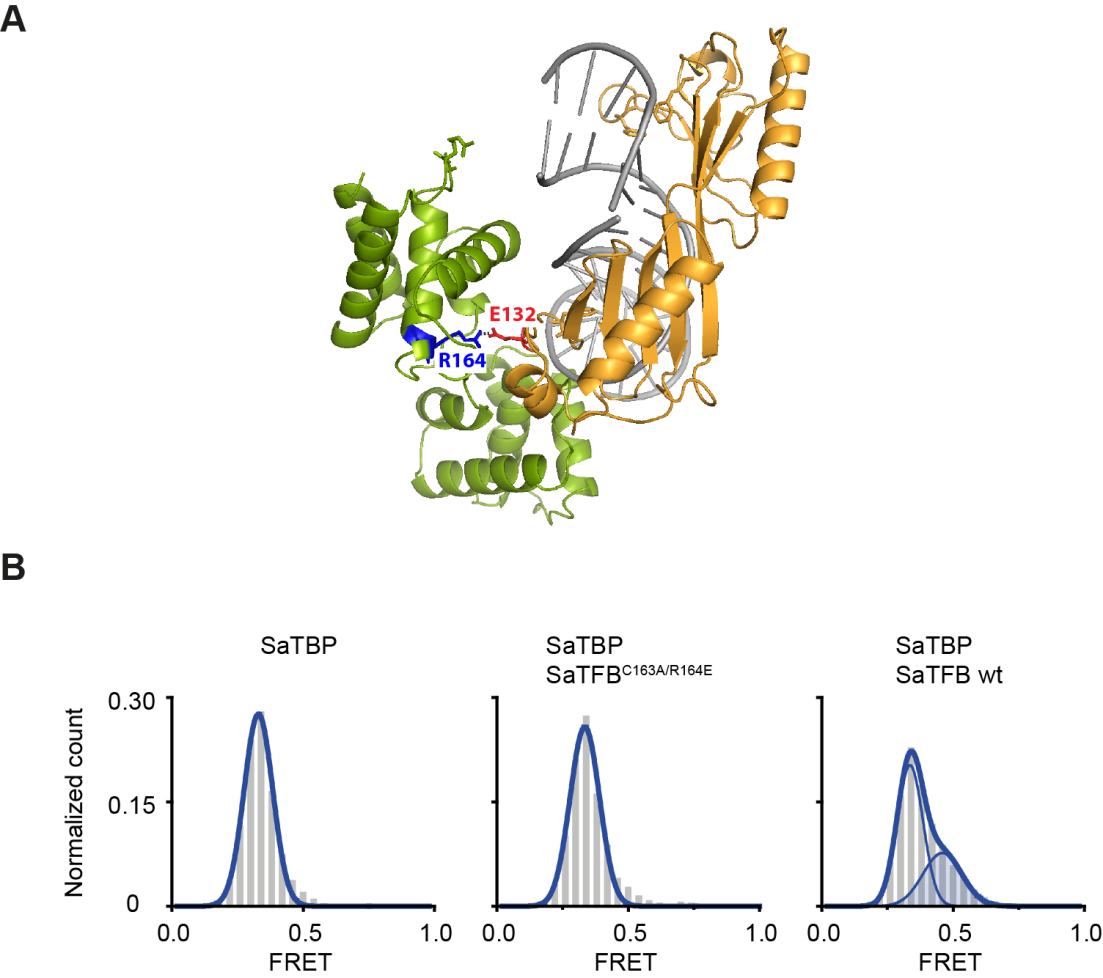


Supplementary Figure S6 related to Figure 2.

**Experiments using a SaTFB mutant. (A)** Ternary complex of TBP (orange), TFB (light green) and promoter DNA (grey) from *Pyrococcus furiosus* (Pw) (PDB:1AIS). The two highly conserved residues R164 (TFB) and E132 (TBP) form a salt bridge at the TFB-TBP interface. TBP^E132^ is part of the C-terminal stirrup of TBP known to form the interaction interface with TFB ([2-4](#_ENREF_2)). **(B)** Single-pair FRET histograms of DNA_SSV_ in the presence of 6 μM SaTBP, 6 μM SaTBP/ 8 μM SaTFB (C163A/R164E) and 6 μM SaTBP/ 8 μM SaTFB (wt). Mutation of the highly conserved arginine and a neighboring highly conserved cysteine in SaTFB (C163A/R164E) leads to a complete loss of DNA bending capability. This indicates that the interaction between SaTFB and SaTBP is crucial for the bending activity.

# Single-molecule titration of transcription factors and promoter DNA


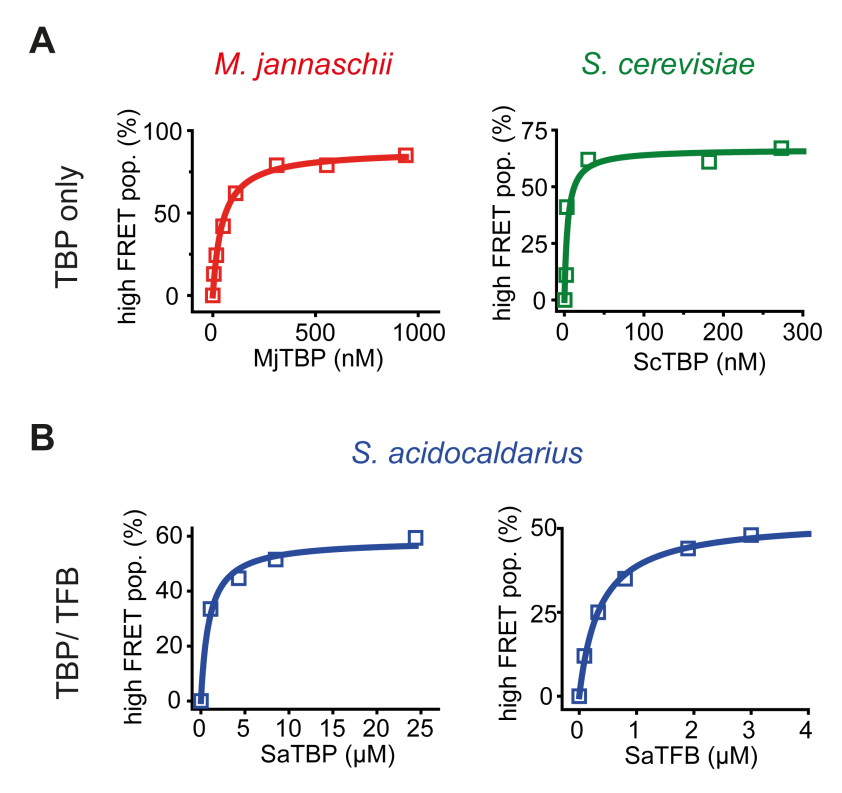


Supplementary Figure S7

**Titrations** **of DNA-TBP and DNA-TBP-TFB interactions**. Experiments were performed using single molecule burst analysis on a confocal microscope. **(A)** The population of the high FRET state (DNA bent conformation) as function of TBP concentration. MjTBP titration revealed a *K*_D_ of 48 ± 6 nM and the titration with ScTBP a *K*_D_ of 4.2 ± 1.2 nM. **(B)** The population of the high FRET state (bent DNA conformation) as a function of SaTBP and SaTFB concentration. In the presence of 3 µM SaTFB, the titration with SaTBP yielded a *K*_D_ of 930 ± 230 nM and, in the presence of saturating SaTBP concentrations (14 µM), the dissociation constant of SaTFB was *K*_D_ = 363 ± 34 nM.

All assays were performed under the same conditions / concentrations as for Figure 2.

# Influence of NaCl concentration on TBP-induced bending of promoter DNA


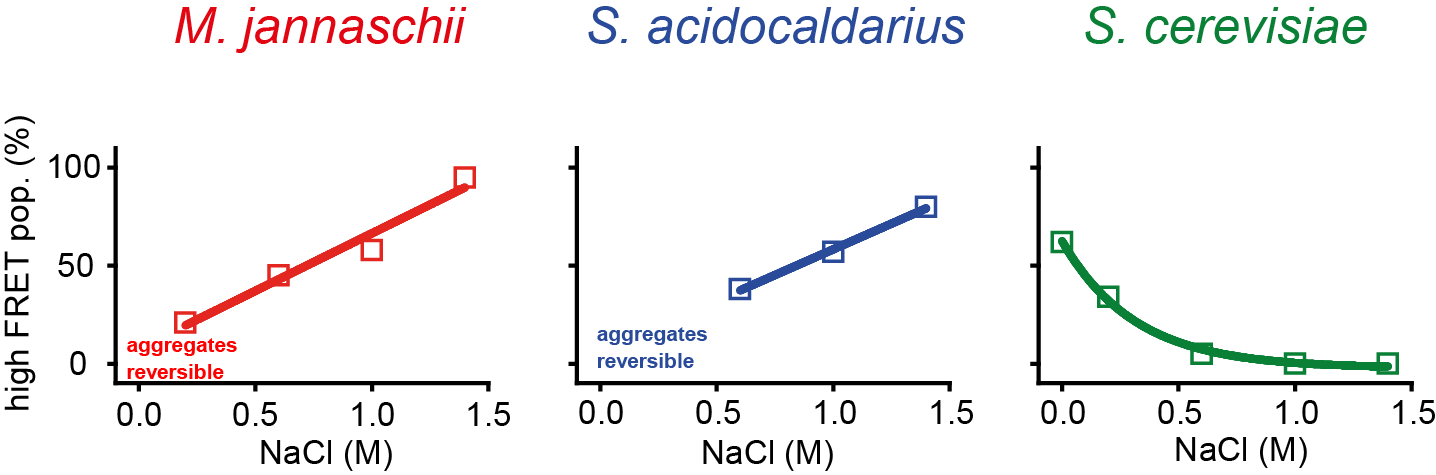


Supplementary Figure S8 related to Figure 3**.**

**Influence of sodium chloride concentration on the TBP-induced bending of promoter DNA.** Measurements were performed using confocal solution measurements at 45°C (archaeal proteins) or room temperature (*S.cerevisiae*). An increase in salt concentration results in a higher fraction of bent DNA when MjTBP and SaTBP/TFB was used. Using NaCl concentrations below 250 mM and 500 mM NaCl, respectively, leads to aggregate formation in the case of the *M.jannaschii* and *S.acidocaldarius* system. In contrast, increasing NaCl concentrations lead to a decrease in the bent DNA fraction for the *S.cerevisiae* system. The lines in all graphs are only present as a guide to the eye.

All assays were performed under the same conditions / concentrations as for Figure 2.

# Representation of the surface charge of the TBP proteins


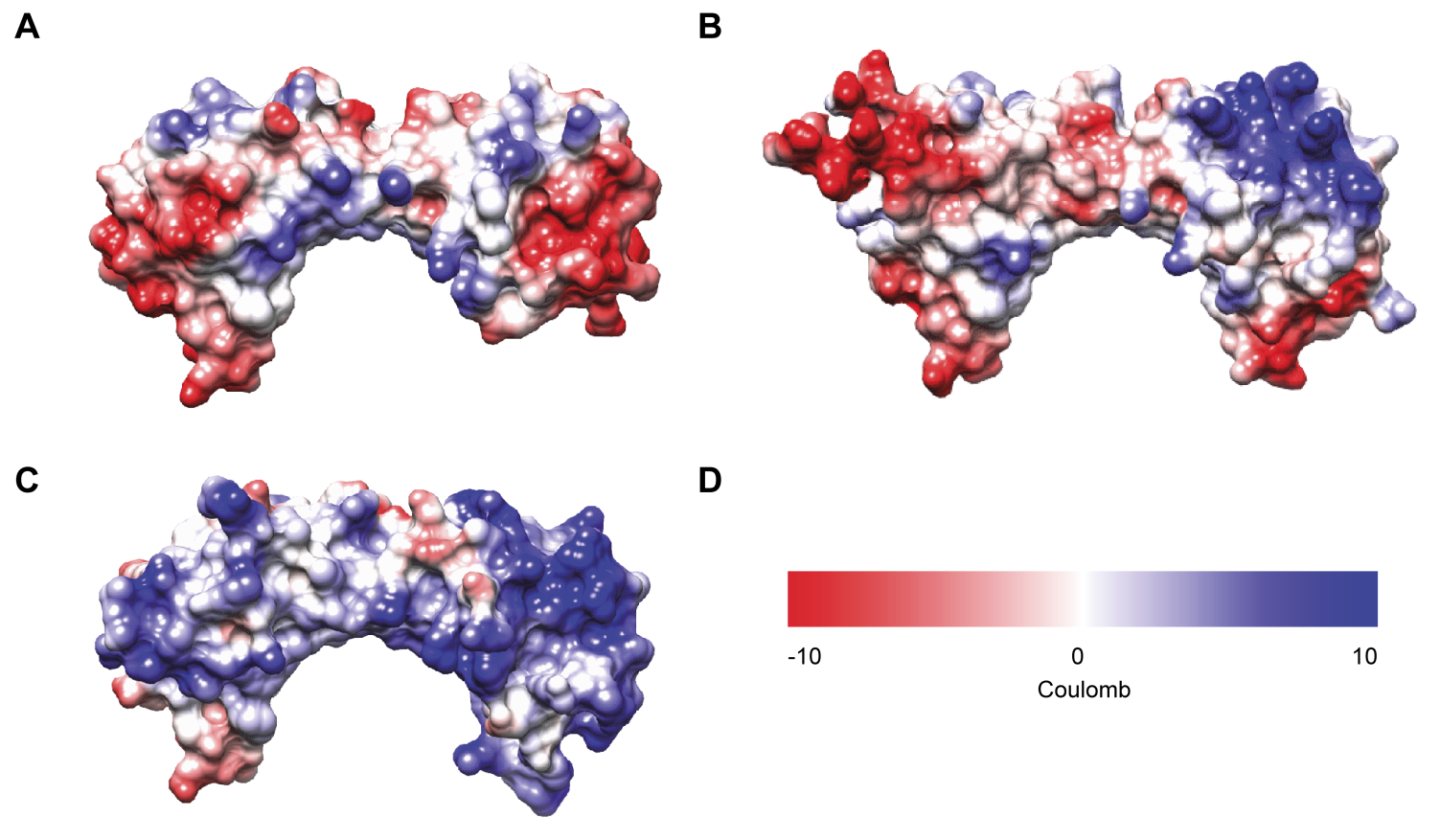


Supplementary Figure S9 related to Figure 3.

**Surface charge density of TBP.** Representation of the surface charge density of TBP from **(A)** *Methanocaldococcus jannaschii* (34 negatively and 23 positively charged amino acids in total), **(B)** *Sulfolobus acidocaldarius* (29 negatively and 28 positively charged amino acids in total) and **(C)** *Saccharomyces cerevisiae* (29 negatively and 39 positively charged amino acids in total). **(D)** The color scale is shown with negatively charged surfaces in red and positively charged surfaces in blue.

# *S.cerevisiae* TIRF data and transition state calculation


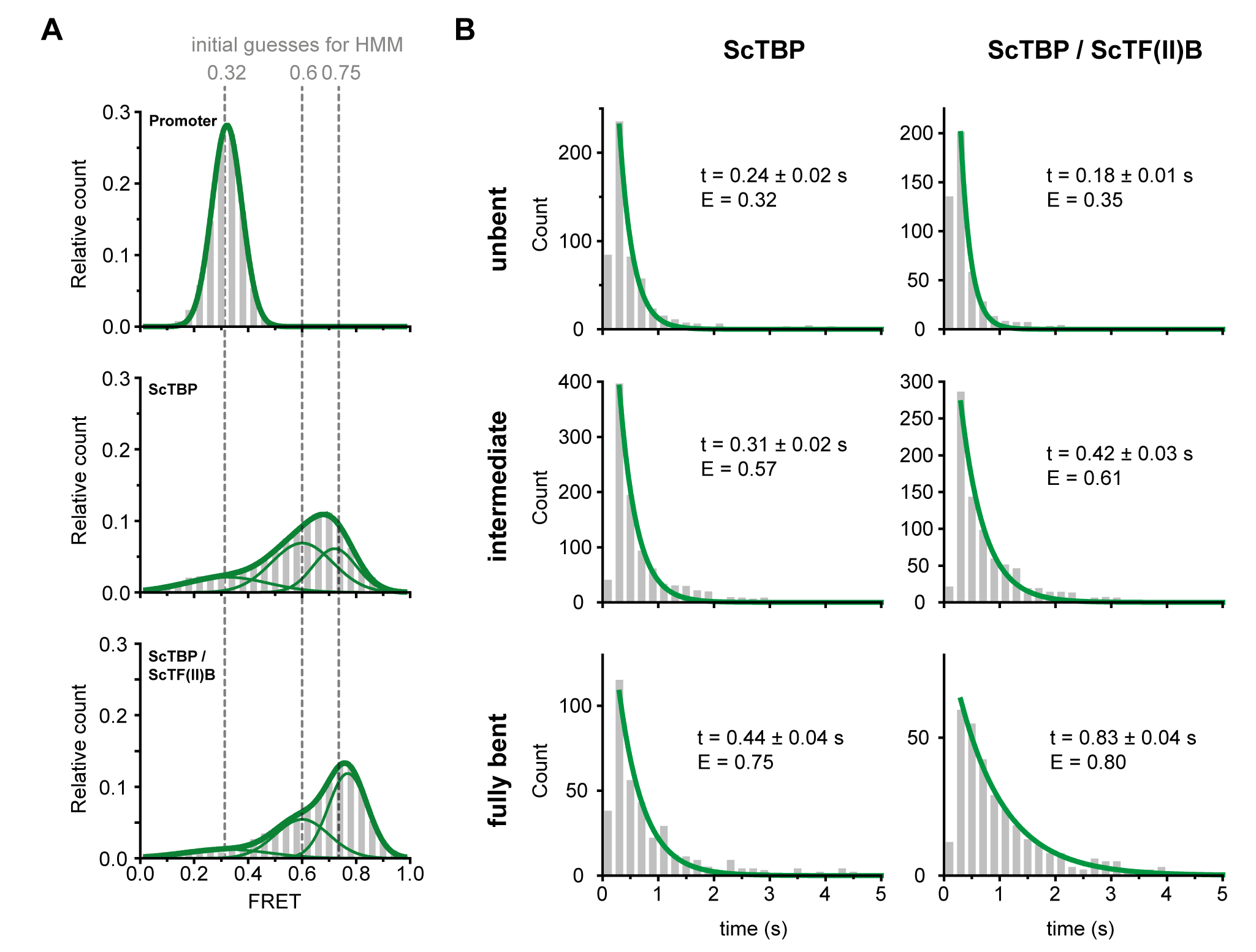


Supplementary Figure S10 related to Figure 5.

**Analysis of ScTBP-DNA interactions using TIRF microscopy. (A)** Framewise FRET histograms of promoter DNA alone (100 molecules), after incubation with 20 nM ScTBP (100 molecules) and after additional incubation with 500 nM ScTF(II)B (100 molecules). The Gaussian fits provide the initial guess for the Hidden-Markov-Model analysis (E_unbent_ = 0.32, E_intermediate_ = 0.6 and E_fully bent_ = 0.75). **(B)** Dwell-time histograms determined from the Hidden-Markov-Model analysis are shown. The dwell times were fitted using a monoexponential and the state’s lifetime and the underlying FRET value are presented in each panel. Details regarding the analysis procedure are given in **Supplementary Figure 13**.

**Transition rate calculation for a linear 3-state model**

We assume a three state system with an unbent state (S_1_), an intermediate state (S_2_) and a fully bent state (S_3_) with transition rates k_mn_ for transitions from state S_m_ to S_n_. Because we only rarely observe direct transitions between states S_1_ and S_3_, we consider these transitions to be artifacts of the limited temporal resolution of the measurement with an EM-CCD camera. Therefore, we consider a linear bending model with only four relevant rates k_12_, k_21_, k_23_ and k_32_ as depicted in Figure 6 of the main text.

From time-resolved TIRF measurements, the dwell time t_n_ for each state S_n_ is calculated (see **Supplementary Figure 10**) as well as the fraction of time spent in either of the states P(n).The dwell times of the states S_1_ and S_3_ can be directly used to determine the sole rate depopulating these states:

$$k_{12}=\frac{1}{t_{1}}$$

$$k_{32}=\frac{1}{t_{3}}$$

In order to determine the two remaining rates, we calculate the steady-state distribution expected from the presented model. The steady-state distribution (time-independent equilibrium) is described by the Eigenvector to the Eigenvalue λ=0 of the set of differential equations

$$\frac{d}{\mathrm{dt}}\left( \begin{matrix} S_{1}(t) \\ S_{2}\left( t \right) \\ S_{3}(t) \end{matrix} \right)=\left( \begin{matrix} -k_{12} & k_{21} & 0 \\ k_{12} & -k_{21}-k_{23} & k_{32} \\ 0 & k_{23} & -k_{32} \end{matrix} \right)\left( \begin{matrix} S_{1}(t) \\ S_{2}\left( t \right) \\ S_{3}(t) \end{matrix} \right)$$

Under the condition that P(1)+P(2)+P(3)=1, we obtain the equilibrium distribution

$$\left( \begin{matrix} P(1) \\ P\left( 2 \right) \\ P(3) \end{matrix} \right)=\left( \begin{matrix} S_{1}(t\to\infty) \\ S_{2}\left( t\to\infty\right) \\ S_{3}(t\to\infty) \end{matrix} \right)=\frac{1}{k_{21}k_{32}+k_{12}k_{23}+k_{12}k_{32}} \left( \begin{matrix} k_{21}k_{32} \\ k_{12}k_{32} \\ k_{12}k_{23} \end{matrix} \right)$$

Using these equations and the previously determined rates k_12_ and k_32_, the rates k_21_ and k_23_ are calculated. Finally, the obtained transition rates from state S_2_ are compared to the actual number of transitions to state S_1_ and S_3_ for consistency:

$$\Phi_{k}=\frac{k_{21}}{k_{21}+k_{23}}$$

$$\Phi_{N}=\frac{N_{21}}{N_{21}+N_{23}}$$

| sample | k_12_ / s^-1^ | k_32_ / s^-1^ | k_21_ / s^-1^ | k_23_ / s^-1^ | _k_ | _N_ |
| --- | --- | --- | --- | --- | --- | --- |
| ScTBP | 4.2 | 2.3 | 1.6 | 1.2 | 0.58 | 0.58 |
| ScTBP + ScTF(II)B | 5.6 | 1.2 | 1.4 | 0.9 | 0.61 | 0.58 |

# Supplementary Methods

## Electrophoretic mobility shift assays (EMSA)

Complementary DNA strands of the SSV T6 promoter were annealed by incubation for 5 minutes at 95°C and slowly cooled down to room temperature. Either a radiolabelled or fluorescently labeled version of the m3 DNA derived from the SSV T6 promoter DNA was used as described earlier ([5](#_ENREF_5),[6](#_ENREF_6)).

The reaction components of the *Methanocaldococcus jannaschii* system indicated in the figure legends were combined on ice in 1x TNME buffer (40 mM Tris/HCl, pH 7.3, 250 mM sodium chloride, 2.5 mM magnesium chloride, 0.1 mM EDTA, 5% glycerol, 10 mM dithiothreitol (DTT)), incubated for 20 minutes at 65˚C and separated on a 12% native Tris-Glycine at 200 V for 45 minutes at room temperature. The final concentrations were as follows: [TBP] = 5.2 µM, [TFB] = 6.7 µM, [DNA_SSV_] = 667 nM, [Heparin] = 400 µg/ml. The non-template strand carried a fluorescence label (Alexa555) at position +17 ([5](#_ENREF_5)) and the DNA was detected using a FLA Scanner (GE Healthcare).

For electrophoretic mobility shift assays using TBP and TFB from *Sulfolobus acidocaldarius,* 15 μl samples containing 0.1 μM TFB and 0.5 μM TBP in 10 mM MOPS pH 6.5, 180 mM sodium chloride, 20 mM potassium acetate, 11 mM magnesium chloride, 10% glycerol, 5 mM DTT, 1 μg BSA, 100 μg/ml Heparin and, 125 fmol of ^32^P 5’end-labelled double-stranded DNA template encompassing the viral SSV1 T6 promoter were used. Samples were incubated at 65°C for 5 min. For supershift experiments, 1 µl of antisera was added and the samples were incubated for 15 min at room temperature. 10 μl were loaded on a 6% continuous native Tris-Glycine gel containing 2.5% glycerol and 0.5 mM DTT. The gel was dried and labeled DNA was detected by phosphorimaging using a FLA Scanner (GE Healthcare).

## Installation and calibration of the heating unit at the TIRF microscope


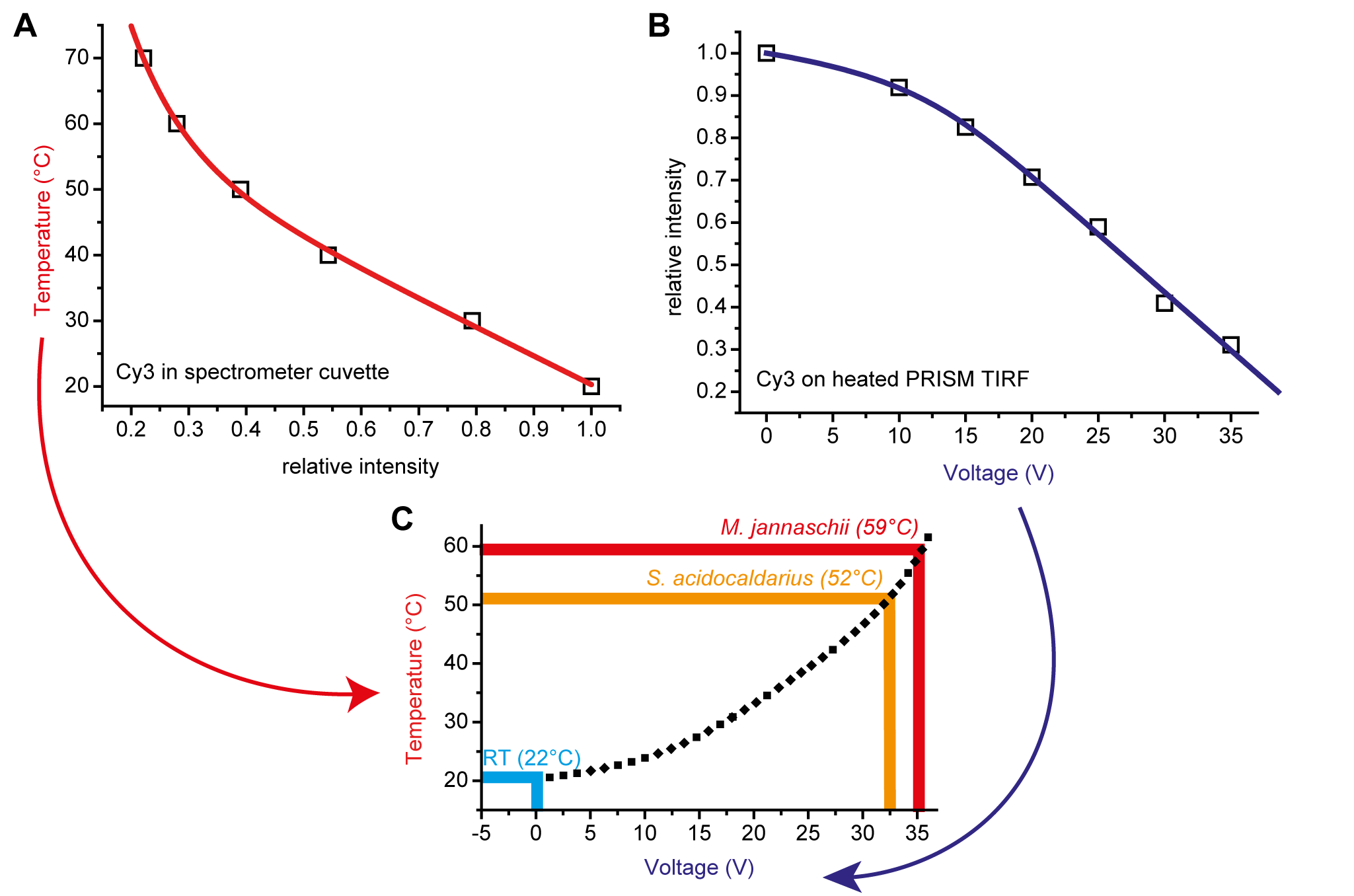


## **Supplementary Figure S11**

**Calibration of the sample temperature on the TIRF microscope**. The temperature sensitivity of the fluorescence intensity of Cy3 was used to calibrate the temperature of the sample on the TIRF microscope. **(A)** 20 nM of a Cy3-labeled single-stranded DNA were heated up with a temperature controlled fluorescence spectrometer (Varian Cary Eclipse) in a fully filled and sealed Hellma cuvette (1.6 ml). The fluorescence intensity was measured every 10°C and the relative intensity decrease was calculated. The data was fitted with a bi-exponential decay (R^2^>0.99). **(B)** The same oligonucleotide was immobilized via biotin-neutravidin linkage on a PEG surface and heated from the top via a peltier element (HK5163R157L12A from MINCO, Minnesota USA) that was glued (J-B WELD “AutoWELD”) to the prism. The peltier element itself was heated up to over 120°C. The prism reached temperatures up to 80°C while the downside of the flow chamber remained cooler than 50°C so that any harm to the water objective due to temperature was avoided. The graph shows the decreasing intensity of Cy3 with the increasing voltage applied to the heating film. Again, the data was fitted to a biexponential (R2>0.99). **(C)** The calibration plot generated by combining the data from the ensemble and single molecule measurements. The three mainly used voltages are indicated: Blue: no voltage applied – room temperature. Orange: 32.5 V applied, 52°C. This was the maximum temperature at which the biotin-neutravidin complex was stable in measurement buffer for *S. acidocaldarius* (pH 6.5 1 M NaCl). Red: 35V applied - 60°C. This was the maximum temperature at which the biotin-neutravidin complex was stable for at least 30 minutes in measurement buffer for *M. jannaschii* (pH 7.0 1 M NaCl) until thermally denaturing. Since the heating is not feedback controlled, a temperature deviation of +/-3°C is assumed.

## Confocal Analysis

A burst analysis experiment was performed on a custom build confocal microscope with dual color detection and alternating laser excitation ([7](#_ENREF_7),[8](#_ENREF_8)). (For details, see the Method section of the main text.) The photon stream was analyzed using a burst search algorithm with following parameters:

Cy3b/ATTO647n: T = 500 µs, M = 30, N = 120
ATTO532/ATTO647n: T = 500 µs, M = 10, N= 50

In some cases, for the *S.cerevisiae* measurements, we applied the FRET-2CDE filter ([9](#_ENREF_9)) as well, allowing only bursts that are within the range from 8 – 12.

For the ScTBP half-life analysis (see Figure 5D in the main text), we used the ALEX-2CDE filter (range: 0 - 12) as well as the FRET-2CDE filter (range: 8 - 12) to eliminate the dominant donor only population (caused by the ATTO532 labeled protein) and the (over time) increasing acceptor only population (caused by the unbound ATTO647n labeled promoter DNA). The remaining FRET population after filtering was normalized for all bursts detected. Its relative change was plotted versus time (2.5 min binning for the first two data points and 5 min binning for rest) and fitted with a monoexponential decay.


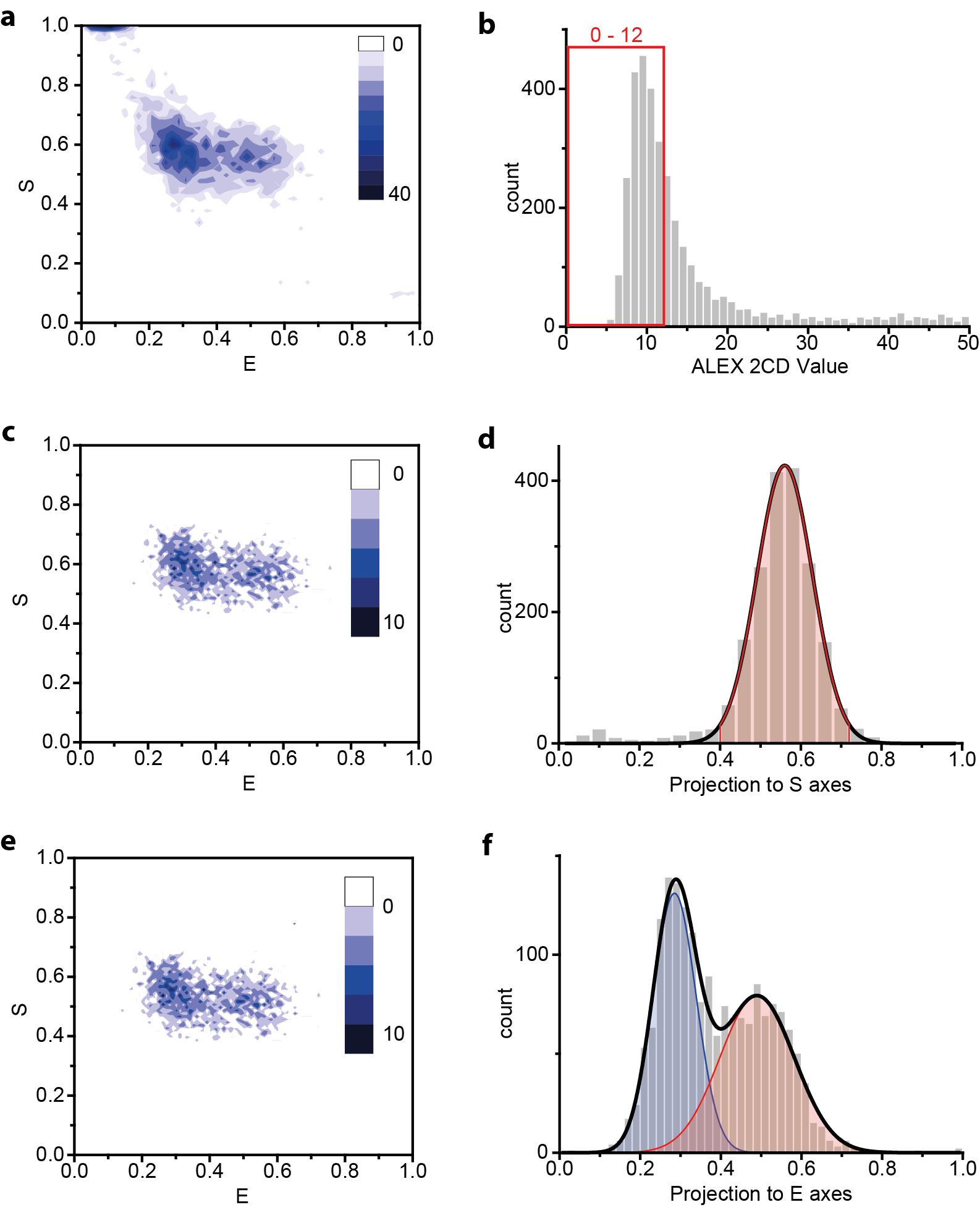


## **Supplementary Figure S12**

**Analysis of the confocal single-molecule burst experiments. (A)** E-S-histogram of the bursts detected by the burst search algorithm for measurements on MjTBP – promoter DNA interaction ([10](#_ENREF_10)). **(B)** A filter for brightness fluctuations within the individual bursts is applied and only bursts with minimal fluctuations (here: ALEX-2CDE < 12) are selected ([9](#_ENREF_9)). **(C)** The E-S-histogram from panel A after filtering. **(D)** The S value distribution is narrowed by only using the bursts with a S-value within 2x FWHM (red shaded area). **(E)** Resulting E-S-histogram from panel C after filtering based on stoichiometry. **(F)** Histogram projected onto the X-axes (FRET efficiency, E). The E-value distribution was fitted with two Gaussian distributions (in the case of ScTBP + ScTF(II)B, three Gaussians were used). The fraction of the areas under each peak, R = A_high FRET_ /(A_low FRET_ + A_high FRET_), can be calculated and plotted against the varying parameter as in Figure 3.

## TIRF analysis


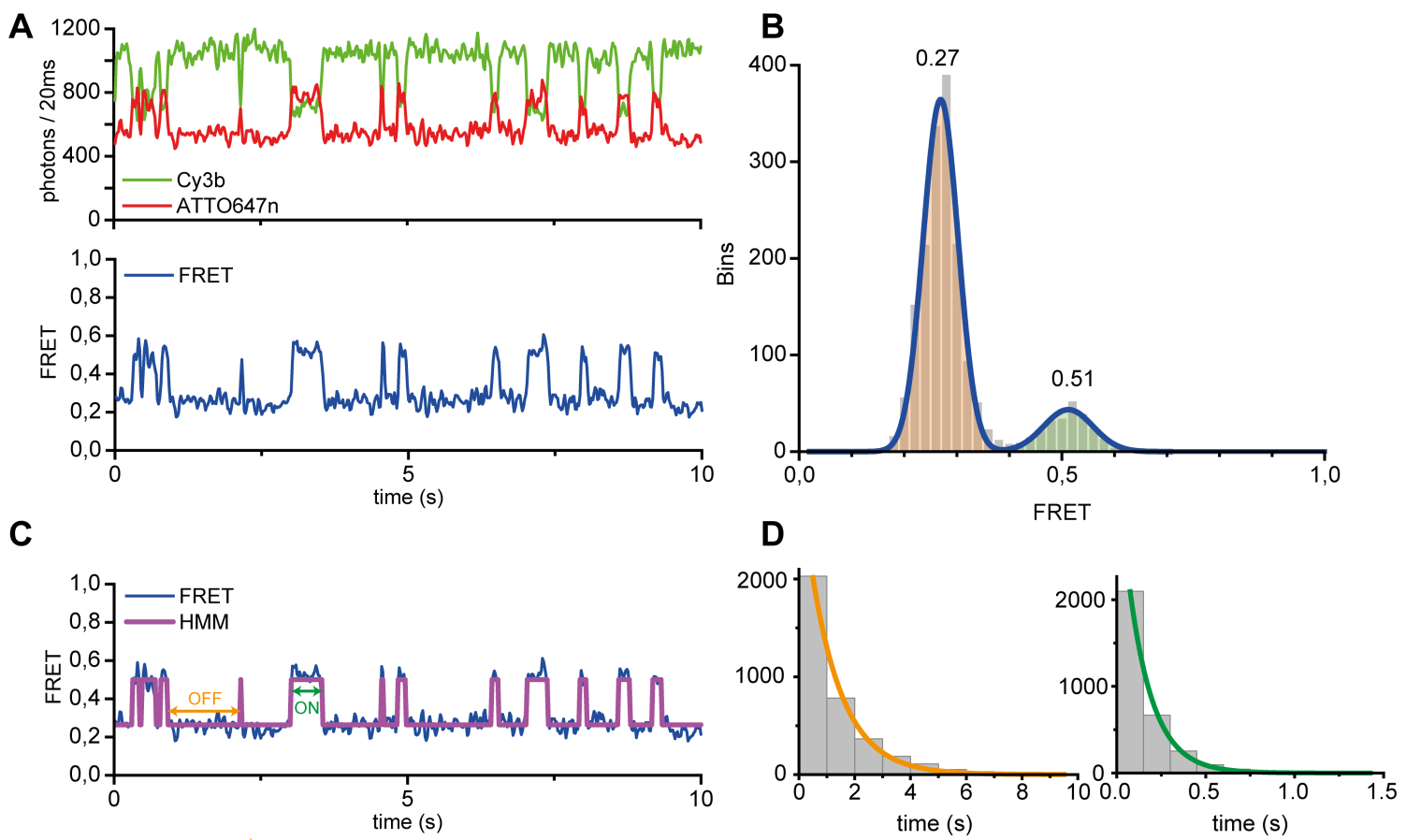


## **Supplementary Figure S13**

**HMM Analysis of single-molecule TIRF data**. **(A)** Upper panel: Typical fluorescence intensity traces of the donor (Cy3b in green) and acceptor (Atto647n in red) molecules showing the transient induced bending events of MjTBP. The donor and acceptor signals are anti-correlated. Lower panel: The uncorrected FRET efficiency (or proximity ratio) E = I_Acceptor_ / (I_Acceptor_ + I_Donor_) is calculated for each frame and plotted as a function of time. **(B)** A framewise histogram of the trace in panel A fitted with two Gaussian distributions (in case of ScTBP / ScTF(II)B three Gaussians were used). The relative areas R=A_high FRET_/(A_low FRET_ + A_high FRET_) provides analogous information to the confocal measurements but for a single molecule. **(C)** The FRET trace shown in panel A (blue) along with the results of the Hidden-Markov-Model (HMM) analysis (purple). The peaks of the Gaussian fit are used as “initial guesses” for the HaMMy analysis ([11](#_ENREF_11)). From the HMM analysis, the OFF (unbent) and ON (bent) dwell times for each event in every transient are determined. For analysis of the ScTBP/ScTF(II)B traces, three states (unbent, intermediate, fully bent) with values of 0.35, 0.6, 0.75 were provided as initial guess for the three-state HMM analysis. **(D)** Histograms of the ON and OFF dwell times for MjTBP on DNA_SSV_ from the HMM analysis. Each state’s dwell time histograms were fit with a mono-exponential. In order to exclude artifacts due to the limited time resolution, dwell times smaller than 3x the integration time of the camera (20 ms for MjTBP, 100 ms for SaTBP, 50 ms for ScTBP) were neglected. The decay time together and standard error for each state are plotted in Figure 5C.

# Supplementary References

1. Zhang, J., Li, E. and Olsen, G.J. (2009) Protein-coding gene promoters in Methanocaldococcus (Methanococcus) jannaschii. *Nucleic Acids Res*, **37**, 3588-3601.

2. DeDecker, B.S., O'Brien, R., Fleming, P.J., Geiger, J.H., Jackson, S.P. and Sigler, P.B. (1996) The crystal structure of a hyperthermophilic archaeal TATA-box binding protein. *J Mol Biol*, **264**, 1072-1084.

3. Cox, J.M., Hayward, M.M., Sanchez, J.F., Gegnas, L.D., van der Zee, S., Dennis, J.H., Sigler, P.B. and Schepartz, A. (1997) Bidirectional binding of the TATA box binding protein to the TATA box. *Proc Natl Acad Sci U S A*, **94**, 13475-13480.

4. Bell, S.D. and Jackson, S.P. (2000) The role of transcription factor B in transcription initiation and promoter clearance in the archaeon Sulfolobus acidocaldarius. *J Biol Chem*, **275**, 12934-12940.

5. Grohmann, D., Nagy, J., Chakraborty, A., Klose, D., Fielden, D., Ebright, R.H., Michaelis, J. and Werner, F. (2011) The initiation factor TFE and the elongation factor Spt4/5 compete for the RNAP clamp during transcription initiation and elongation. *Mol Cell*, **43**, 263-274.

6. Werner, F. and Weinzierl, R.O. (2005) Direct modulation of RNA polymerase core functions by basal transcription factors. *Mol Cell Biol*, **25**, 8344-8355.

7. Kapanidis, A.N., Lee, N.K., Laurence, T.A., Doose, S., Margeat, E. and Weiss, S. (2004) Fluorescence-aided molecule sorting: analysis of structure and interactions by alternating-laser excitation of single molecules. *Proc Natl Acad Sci U S A*, **101**, 8936-8941.

8. Muller, B.K., Zaychikov, E., Brauchle, C. and Lamb, D.C. (2005) Pulsed interleaved excitation. *Biophys J*, **89**, 3508-3522.

9. Tomov, T.E., Tsukanov, R., Masoud, R., Liber, M., Plavner, N. and Nir, E. (2012) Disentangling subpopulations in single-molecule FRET and ALEX experiments with photon distribution analysis. *Biophys J*, **102**, 1163-1173.

10. Nir, E., Michalet, X., Hamadani, K.M., Laurence, T.A., Neuhauser, D., Kovchegov, Y. and Weiss, S. (2006) Shot-noise limited single-molecule FRET histograms: comparison between theory and experiments. *J Phys Chem B*, **110**, 22103-22124.

11. McKinney, S.A., Joo, C. and Ha, T. (2006) Analysis of single-molecule FRET trajectories using Hidden Markov Modeling. *Biophys J*, **91**, 1941-1951.
